# Unveiling the mechanistic nexus: how micronutrient enrichment shapes brain function, and cognitive health

**DOI:** 10.3389/fmolb.2025.1623547

**Published:** 2025-09-23

**Authors:** Siddhartha Das, Pradipta Banerjee, Sudipta Jana, Hemanshu Mondal

**Affiliations:** ^1^ Department of Plant Pathology, M. S. Swaminathan School of Agriculture, Centurion University of Technology and Management, Paralakhemundi, Odisha, India; ^2^ Department of Surgery, University of Pittsburgh, Pittsburgh, PA, United States; ^3^ Department of Biochemistry, M. S. Swaminathan School of Agriculture, Centurion University of Technology and Management, Paralakhemundi, Odisha, India; ^4^ Syngenta PVT Ltd., Ranchi, Jharkhand, India

**Keywords:** cognitive decline, brain development, neuroplasticity, epigenetics, mental health

## Abstract

Minerals, vitamins, and trace elements are examples of micronutrients essential for psychological wellbeing and brain function. Severe disorders may result from their deficiency or, conversely, from an excess of them. Recent studies have indicated that the etiopathogenesis of certain neurological disorders may involve chronically elevated micronutrient levels. Physiological functions, such as energy metabolism, neurotransmitter synthesis, and antioxidant defence, are regulated by these vital nutrients and are essential for optimal neuronal activity. According to new research, micronutrient enrichment, whether through diet or supplements, can have a significant impact on cognitive function, neuroplasticity, and brain development. Cognitive decline, memory loss, and attention problems are linked to deficiencies in essential micronutrients, including vitamin B12, iron, zinc, and omega-3 fatty acids. Tailored micronutrient therapies have shown promise in reducing age-related cognitive decline and enhancing mental function in both healthy individuals and those at greater risk. This manuscript emphasizes the growing research linking micronutrient status to cognitive health. It also highlights the importance of maintaining a balanced diet and following appropriate supplementation practices to optimize brain function throughout life.

## 1 Introduction

The intricate connection between diet and brain development has long captivated scientists with an increasing emphasis on the role that micronutrients play in affecting cognitive performance. While there is an acknowledgment of the importance of various micronutrients in brain health, the specific biochemical mechanisms through which they exert their effects on cognitive function are still not well understood. More research is needed to elucidate how different micronutrients interact on a molecular level with brain cells. Much of the existing research is cross-sectional, which limits the understanding of the long-term effects of micronutrient enrichment on cognitive health. Longitudinal studies would provide insight into how sustained micronutrient intake influences cognitive decline over time. Micronutrients, which include minerals, vitamins, and trace elements, are vital cofactors in biochemical processes that are essential to brain development, neuroplasticity, and neuronal function (Adan et al., 2019). Recent developments in epigenetics and nutritional neuroscience have revealed an intriguing intersection where micronutrient enrichment can affect synaptic activity, neuronal signaling pathways, and gene expression. Are they what we consume? Do the foods we eat affect our vulnerability to illnesses, including mental health conditions? Two significant elements influence a person’s vulnerability to mental health issues throughout the lifespan: environmental and hereditary (Alexander Arguello et al., 2019; Adams, 2019). Mental health disorders are becoming more common, which has a significant effect on both the afflicted individuals and society as a whole. As a metabolically active organ, the brain relies on a consistent supply of specific micronutrients to support oxidative defense, neurotransmitter synthesis, and energy metabolism. Deficits in essential micronutrients, such as iron, zinc, folate, and omega-3 fatty acids, have been linked to accelerated neurodegeneration, neurodevelopmental disorders, and impaired cognitive performance (Mattei and Pietrobelli, 2019). Most of the research often examines individual micronutrients in isolation. Investigating the synergistic effects of multiple micronutrients and their combined impact on brain health is an area that requires further exploration. However, dietary enrichment or specific alternatives have demonstrated potential for enhancing resilience to stress, optimizing cognitive function, and delaying age-related cognitive decline.

As per the World Health Organization (WHO), around 970 million individuals worldwide, or one in eight people, suffer from mental health issues (World Health Organization, 2024). In 2021, the National Institute of Mental Health (NIMH) estimated that one out of five American individuals suffer from a mental health issue (National Institute of Mental Health, 2024). An additional global public health concern that is on the rise is obesity, which highlights the intricate connection between nutrition, health, and illness. While theoretical frameworks exist, there is a lack of practical guidelines for incorporating micronutrient enrichment into dietary recommendations or interventions designed to improve cognitive health. More studies are needed to translate research findings into actionable health strategies. The World Health Organization’s data indicates that obesity rates have significantly increased since 1990, doubling for adults and quadrupling for adolescents. This alarming trend coincides with an increase in mental health disorders. In 2022, approximately one in eight individuals was estimated to be obese, while over 37 million children, some as young as five, were considered overweight (National Institute of Diabetes and Digestive and Kidney Diseases, 2024; World Health Organization, 2025). These startling figures underscore the urgency of addressing and resolving these health issues. Emerging evidence suggests that the gut microbiome plays a crucial role in nutrient absorption and overall brain health. Research gaps exist in understanding how micronutrient enrichment affects the gut microbiome and, in turn, cognitive functions. These medical disorders have intricate, multifaceted underlying mechanisms. Recent developments in neuroscience and epigenetics show that mental health problems are often associated with environmental factors in addition to genetic ones (De Kloet et al., 2005). These factors include socioeconomic status, early life experiences, levels of physical activity, stress exposure and coping strategies, and diet quality and quantity (Adams, 2019; Dauncey, 2012). Early exposure to these factors may have a lasting impact on both mental health and physiology (Gabbianelli and Damiani, 2018; Ramos Lopez et al., 2021). This review highlights the intricate connection between diet, psychological wellness, and epigenetic alterations. The impact of early nutrition on the interactions and optimum functioning of the cognitive system throughout life has been explained by a broad spectrum of concepts (Klengel et al., 2014). Numerous disorders, including mental health conditions, are believed to be influenced by genes and processes that include DNA methylation, histone modifications, and microRNA activity (Ptak and Petronis, 2010; Nestler et al., 2016). Significant shifts in brain plasticity occur during the early phases of development and are particularly susceptible to environmental influences. The epigenetic landscape of the brain can be significantly altered by these modifications, particularly during the critical phases of development. The micronutrients that are required in modest amounts and serve as methyl-donors in one-carbon metabolism include choline, betaine, folate, both vitamins B6 and B12, with the addition of methionine playing a significant role in brain development and the key topic of this review (Martín-Masot et al., 2020). Furthermore, we will explore how epigenetic modifications during early development influence the expression of key genes, particularly those involved in stress response and metabolic processes. By acting as intermediaries or cofactors in complex one-carbon metabolism biochemical pathways, these methyl-donor micronutrients are involved. Preventing mental health issues and early childhood obesity necessitates knowledge of the early life interactions between nutrients and genes (Wachs et al., 2014). The relationship between nutrition and mental health, as elucidated by dietitians and medical professionals, informs the development of preventive strategies and personalized dietary and lifestyle interventions that cultivate lifelong mental wellbeing. This study examines the mechanistic role of methyl-donor micronutrients and their impact on monomeric metabolism. The effects of these micronutrients on cognitive performance, brain function, and metabolic markers are summarized in various clinical trials involving animal and human samples (Fekete et al., 2023). This evidence underscores the importance of early nutritional interventions in supporting mental health, highlighting the crucial need to integrate nutritional science into personalized healthcare from a young age. To understand how micronutrient enrichment affects brain health, this paper examines the epigenetic mechanisms involved. Our research investigates the mechanisms by which micronutrients impact cognitive outcomes by influencing gene-environment interactions. Specifically, we will investigate how micronutrients participate in epigenetic processes, such as DNA methylation, non-coding RNA regulation, and histone modification, thereby modulating gene expression. Gaining knowledge of this relationship can help develop new Nutritional approaches to promote mental wellbeing and mitigate the effects of cognitive deficits across all age groups. There is a need for more focused research on how micronutrient enrichment during critical periods of brain development (such as prenatal and early childhood stages) influences long-term cognitive outcomes.

### 1.1 Why micronutrients are necessary for cognitive development

This review examines the current understanding of micronutrient involvement in various neurological illnesses. The vitamins and trace elements that make up this group of micronutrients are extremely rare in the human body (Vitamin and trace element plasma levels range from mol/L to mg/L, whereas trace element levels can exceed 50 mg/kg), but they are crucial for several allied processes (Berger et al., 2022). One of the few, if any, vitamins that the human body can synthesis is vitamin D. A shortage in fat soluble vitamins may not be apparent for a longer time because the body accumulates lipid soluble vitamins (A, D, E and K) but not water-soluble vitamins (B and C) (Stevens et al., 2022; Plantone et al., 2023). Depletions resulting from inadequate intake or loss of micronutrients can have clinical and laboratory repercussions.

## 2 Methods

### 2.1 Data acquisition strategy

A thorough literature analysis was conducted using databases such as Google Scholar, Web of Science, Scopus, and PubMed, following the suggested reporting items for systematic studies and Meta-analyses (PRISMA) criteria. The search encompassed publications from 2004 to 2025. The search incorporated relevant English keywords, including micronutrients and cognitive health, micronutrients and brain development, micronutrients and gene regulation in brain development, types of micronutrients in both brain and mental health, and types of micronutrients and microbial associations for brain development and cognitive health, among others. The sources for similar meta-analyses and systematic reviews that examined the role of micronutrients in associative gene regulation, mental health, and brain development. The search covered items published up until March 2025 and was not limited by language.

### 2.2 Selection criteria/parameters consideration

The study’s publications focused on how various micronutrients, gene controls, and associated microbiota influence brain development. Additionally, the articles also explored crucial micronutrients, including their doses, mechanistic approaches, gene regulations, and synergistic and antagonistic effects, among others. The exclusion criteria included synthetic micronutrients, macronutrients, growth retardants, toxicological effects of micronutrients, gut microbiota, gut health, over-intake, magnification, and carcinogenic effects. To ensure the integrity of the review, identical replicated research and publications from the same author were excluded. We also disregarded articles that did not present novel or updated methodologies. We independently screened abstracts and titles using predetermined standards for inclusion and exclusion, and obtained full-text copies of potentially relevant studies. Furthermore, we hand-searched the references of retrieved papers to identify additional relevant publications. Discrepancies in reviewer assessments were resolved through debate and agreement.

### 2.3 Data extraction and analysis

*In vivo* mechanistic approaches of micronutrients for brain development and cognitive health, along with genetic regulation, synergistic and antagonistic effects with other micronutrients, and aligned information acquired for these studies. The initial article was expanded to include information on daily intake, the importance of micronutrients in preventing various neurological illnesses, and their requirements at different stages of life.

### 2.4 Qualitative assessment in terms of bias confirmation

The randomization procedure, any departures from the intended treatments, the method for measuring outcomes, and the selection of results to report are the five main areas that must be examined to evaluate overall bias. The GRADE scale, which stands for Grading of Recommendations, Assessment, Development, and Evaluation (GRADE) scale. (https://gdt.gradepro.org).

## 3 Results

The first search revealed a total of 14,272 items with the potential keyword “Micronutrients and cognitive health.” A similar search using “micronutrients” and “brain development” yielded 15,269 items. Searching with the more relevant phrase’ Micronutrients association and Gene regulation on brain development’ generates 5,916 entities. A restriction search from 2004 to 2025 with the keyword entity “Types of micronutrients in both brain and cognitive health” generates 4,399 entities. Align search entitled “Types of micronutrients and microbial association for brain development and cognitive health” produced 1,263 results. Following a rigorous review of the titles and abstracts, articles were selected for further investigation. After completing the comprehensive content analysis, 1,148 of these items (91%) were deleted because they did not meet the accurate goal-setting criteria (the criteria for removal are specified in the Methods section). Out of 115 considered materials, the final comprehensive synthesis, which totals 61 entities (53%), is presented under this investigation.

### 3.1 Food-derived micronutrients

Micronutrients derived from food, such as iron, can be found in leafy greens and red meat. Nuts and shellfish contain zinc, flaxseeds and fatty fish have omega-3 fatty acids, and vitamins like folate (found in citrus fruits) and B12 (found in dairy) are essential for brain function (Clemente-Suárez et al., 2025). They are crucial for neurogenesis, neurotransmitter synthesis, and neuroplasticity, which in turn directly impact cognitive functioning and the ability to resist disorders. A diet rich in nutrients is the first step towards optimal brain function and proper epigenetic regulation. The significance of a varied, nutrient-rich diet in maintaining optimal brain function and cognitive health is highlighted in Table 1.

**TABLE 1:** Lists the micronutrients that are derived from food, their sources, and how they affect cognitive health and brain function.

Micronutrients	Food source	Role in brain function & cognitive health
Iron	Red meat, Spinach, Lentils, Fortified cereals	✓ Supports oxygen transport ✓ Neurotransmitter synthesis ✓ Energy metabolism
Zinc	Shellfish, Nuts, Seeds, Whole grain	✓ Promotes neurogenesis ✓ Synaptic transmission ✓ Antioxidant protection
Vitamin B12	Meat, Fish, Eggs, Dairy	✓ Essential for myelin formation ✓ DNA synthesis ✓ Cognitive function
Vitamin B9 (Folate)	Leafy greens, Beans, Citrus fruits	✓ Key for DNA methylation ✓ Neurodevelopment ✓ Neurotransmitter synthesis
Omega-3 Fatty acid	Fatty fish (Salmon), Flaxseeds, Walnuts	✓ Supports neuroplasticity ✓ Cell membrane integrity ✓ Anti-inflammatory effects
Vitamin D	Fatty fish, Egg yolks, Fortified foods, Sunlight	✓ Regulates brain calcium signaling ✓ Immune responses ✓ Supports mood regulation
Magnesium		✓ Facilitates synaptic plasticity ✓ NMDA receptor’s function ✓ Stress regulation
Vitamin E	Nuts, Seed, Vegetable oil, Spinach	✓ Acts as a neuroprotective antioxidant ✓ Preventing oxidative stress damage
Choline	Eggs, Meat, Fish, Soy products	✓ Crucial for acetylcholine synthesis ✓ Memory & Brain developments
Iodine	Seafoods, Iodized salt, Dairy	✓ Necessary for thyroid hormone production ✓ Rain development
Selenium	Brazil nuts, Seafood, Eggs	✓ Provides antioxidant protection ✓ Supports thyroid hormone metabolism
Copper	Shellfish, Nuts, Seeds, Organ meats	✓ Involved in neurotransmitter synthesis ✓ Iron metabolism

### 3.2 Nutrition and brain development

The brain develops in early adulthood and childhood, according to brain imaging. Moreover, the development and maturation paths of various brain structures vary (Lebel et al., 2012). The brain’s development is influenced by genetic predisposition. Brain function is significantly impacted by early experiences, resulting in individual differences that could increase the chance of developing chronic illnesses throughout one’s lifetime and cause behavioral dysfunction (Miguel et al., 2019). In addition to the environment a child is exposed to, nutrition plays a crucial role in modulating early brain development. In addition to widespread undernutrition in macronutrients, deficiencies in specific nutrients can have a significant impact on neurodevelopment and have lasting consequences. Several nutrients are essential for the brain’s growth during pregnancy, including certain fats, protein, iron, copper, zinc, iodine, and folate (NIH, 2011). Because brain development is a continuous process, the need for these nutrients persists into later life (Table 2). Additionally, the brain develops and grows rapidly during infancy, primarily due to the baby’s nutritional intake. There are several ways that breastfeeding can impact cognitive development, both in relation to the breastfeeding experience and the composition of breast milk (Prado and Dewey, 2014). Breastfeeding is associated with higher IQ scores in children and adolescents across all income levels (Victora et al., 2015). Breastfeeding is said to have cognitive benefits that last into adulthood. In the first few months after birth and during fetal development, general malnutrition has detrimental effects on brain development that last a lifetime and causes learning challenges (such as problems with self-control and subpar academic achievement) (Jirout et al., 2019). A steady supply of nutrients is necessary because synapses are rapidly formed in early and middle childhood and selectively removed later in adolescence (Prado and Dewey, 2014). Throughout adolescence, the brain continues to develop, particularly in areas associated with enhanced cognitive function (Black et al., 2017). Evidence suggests that several nutrients are necessary in adulthood to minimize the adverse effects of ageing on the brain and to promote neuroplasticity and brain function (Goyal and Lannotti, 2018).

**TABLE 2:** Important neurodevelopmental processes influenced by particular nutrients Puri et al. (2023).

Cell type	Function	Nutrient example
Neuron	Division, Migration, Synaptogenesis, Neurite outgrowth	Iron, Copper, Zinc, Vitamin A D C, Iodine, Vitamin B_6_ Protein, Carbohydrates
Oligodendrocyte	Myelination	Iron, Zinc, Iodine, Vitamin B_6_ and B_12_ Protein, Selenium, Carbohydrates
Neuron Astrocyte	Neurotransmitter, Reuptake, Receptor, Concentration	Iron, Zinc, Iodine, Vitamin B_6_ and D, Protein, Selenium, Copper

### 3.3 The impact of micronutrients on cognitive health

Protein consumption and overall cognitive function in old age did not significantly correlate, according to the Coelho-Junior et al. meta-analysis (Coelho-Júnior et al., 2021). Nonetheless, three studies have shown that protein consumption and memory are positively correlated. According to a single study, eating protein improves sustained attention, processing, verbal fluency, and visuospatial ability (Okereke et al., 2013). In their cross-sectional investigation, Li et al. found a favorable correlation between consuming eggs for protein, lentils, and other animal products, and cognition (Li X. et al., 2020). A high-fat diet may have a negative impact on cognition, according to studies conducted in adults and senior citizens. A long-term study of 6,18 older women in the US found that lower verbal and cognitive memory trajectories were associated with increased saturated fatty acid content. Conversely, better trajectories were linked to higher MUFA intake (Okereke et al., 2013). According to Francis and Stevenson’s review, reduced cognitive function is associated with a diet heavy in sugar-processed carbs and saturated fat (Francis and Stevenson, 2013). A high-fat diet impairs memory because it triggers a neuro-inflammatory reaction in the hippocampus in response to even a minor immunological challenge (Spencer et al., 2017). Obesity, diabetes, deterioration in cognition, and even Alzheimer’s disease (AD) are all increased by a high-fat diet. Alzheimer’s Disease (AD) risk is elevated by resistance to insulin, impaired metabolism of glucose, and type 2 diabetes mellitus (Barbagallo, 2014). Diets high in protein have inconsistent effects; however, low-fat diets can stop cognitive deterioration. Polyunsaturated fatty acids (PUFAs) play a crucial role in regulating the composition and activity of neurons, Glial cells, and cells of endothelial tissue within the brain. In particular, the omega-3 fatty acids docosahexaenoic acid (DHA) and eicosapentaenoic acid (EPA) influence brain function by modulating neurotransmission, reducing neuroinflammation, and promoting the survival and growth of new neurons (neurogenesis) (Bazinet and Laye, 2014). DHA plays a vital role in brain health by influencing neurotransmitter systems, especially those involved in vision. It is essential for maintaining the proper fatty acid composition and fluidity of cell membranes, and it supports critical brain development processes like synaptogenesis, neurogenesis, and neuronal migration. These processes are vital for brain regions that govern concentration, impulsivity, and inhibition (Cusick and Georgieff, 2016). Furthermore, the anti-inflammatory and anti-thrombotic properties of PUFAs, combined with their positive impact on brain function, contribute to maintaining cognitive function and protecting against dementia (Gillette-Guyonnet et al., 2013). According to a Chinese retrospective investigation, a low intake of energy from fat and protein is associated with a decline in cognitive function in later life (Ding et al., 2018). Memory loss may also result from inadequate intake of Omega-3 polyunsaturated fatty acids (Spencer et al., 2017).

### 3.4 Food groups and dietary patterns

Diet plays a vital role in brain health, influencing both its function and long-term maintenance. Consuming a healthy diet may offer protection against dementia as well as moderate cognitive impairment and mild cognitive impairment. Research by Smyth et al. suggests that prioritizing nutrient-rich foods is a promising method of reducing the widespread problem of cognitive decline (Smyth et al., 2015). Wright et al. showed that, regardless of race or socioeconomic background, improved mental function, particularly through better diets, is linked to enhanced language retention and memory (Wright et al., 2017). The “whole diet approach” theory is a balanced diet that promotes brain health, rather than focusing on a single nutrient—is supported by evidence. Specific dietary patterns, such as the Nordic diet, the DASH diet, or the Mediterranean diet, may be more beneficial than consuming certain foods or food groups.

### 3.5 The Mediterranean diet

The Mediterranean diet encompasses the traditional eating patterns of residents in nations bordering the Mediterranean Sea, including Egypt, Algeria, Libya, Greece, Spain, France, and Italy. This dietary approach emphasizes consuming a variety of vegetables, fruits, cheese, yogurt, and minimally processed carbohydrates and starches. Red meat consumption is limited to a few times per month, whereas fish, chicken, and eggs are eaten a few times every week. Between 28 and 40 percent fat, mainly from unsaturated sources like olive oil, is present (Aridi et al., 2017). This diet has been associated in studies with a lower risk of dementia, Alzheimer’s disease, depression, and cognitive decline (Kesse-Guyot et al., 2013; Samieri et al., 2013; Lourida et al., 2013). In a PREDIMED sub-study, researchers evaluated cognitive function at baseline and 4 years later (Solfrizzi, 2014; Woodside et al., 2014). Cognitive performance improved in subjects who ate a Mediterranean diet, while those following the control diet showed a decline (Valls-Pedret et al., 2012). Long-term studies consistently suggest that following a Mediterranean diet may help protect against cognitive decline and Alzheimer’s disease (Psaltopoulou et al., 2013). Van de Rest et al.'s comprehensive review of numerous cross-sectional studies, longitudinal studies, clinical trials, and meta-analyses implies that increased adherence to a Mediterranean diet is associated with reduced risk or delayed start of cognitive degradation, dementia, and Alzheimer’s disease (van de Rest et al., 2015).

### 3.6 The nordic diet

The foods that people in Scandinavia eat are the basis of the Nordic Diet (Morris, 2017). Fruits, vegetables, fish, canola oil, and various meats are among the foods and nutrients that are highlighted. In a 4-year study, 1,140 individuals with typical cognitive abilities participated in an investigation of the connections between cognitive performance and the Nordic diet. It was found that, compared to the baseline, subjects who adhered to the Nordic Diet’s recommendations showed enhanced cognitive functioning levels (Männikkö et al., 2015). The Dietary Approaches to Stop Hypertension (DASH) diet moderates portion sizes and reduces salt content, providing substantial health benefits. The DASH diet decreased cardiovascular risk variables and was particularly beneficial for subjects with a higher cardiometabolic risk (Siervo et al., 2014). The MIND diet, a variation of the Mediterranean and DASH diets designed to delay neurodegeneration, incorporates brain-healthy recommendations. Specifically, it emphasizes the consumption of antioxidant-rich foods to improve cognitive function, as well as vegetables with green leaves to prevent cognitive decline (Boespflug et al., 2018; Nilsson et al., 2017; Whyte et al., 2018), and blueberries to enhance memory (Morris, 2017). Fish, which contain high levels of EPA and DHA, can also help maintain cognitive function (Ghasemi Fard et al., 2019). Green tea, seaweed, mushrooms, soy, green leafy vegetables, and whole grains are the mainstays of Asian plant-based diets. Research consistently demonstrates that specific dietary patterns are associated with cognitive benefits (Crawford et al., 2021). These benefits include improved logical memory, better performance on overall cognitive assessments, a decreased likelihood of cognitive decline, and a slower rate of cognitive decline. Additionally, Van de Rest et al. (2015) discovered that several healthy diet patterns were gathered through both pre-established techniques, which were also associated with a lower probability of dementia and cognitive decline (like the Healthy Diet indicator and Healthy Eating Index), and data-driven methods (like cluster analysis, factorial analysis, and decline regression model) (Siervo et al., 2014).

### 3.7 Fruits and vegetables

Additionally, research has been done on how specific dietary groups-like nuts, fruits, vegetables, and fermented foods-affect the gut microbiota. Recently, a thorough analysis of the impact of various fruits and vegetables on intestinal flora was published (Han and Xiao, 2020). Eating fruits and vegetables has been demonstrated in numerous studies on humans and animals to increase microbial diversity and function, alter bacterial phylum abundance, lower potentially dangerous bacteria like *E. coli* and *Enterococcus* and stimulate the mass multiplication of beneficial bacteria such as *Lactobacillus* and Bifidobacterium (Paturi et al., 2017; Guglielmetti et al., 2013; Duque et al., 2016; Kaczmarek et al., 2019). The potential health benefits of certain foods may stem from microbiota-accessible carbohydrates (MACs), a group encompassing resistant starches, inulin, pectin, cellulose, oligosaccharides, and lignans. Research by Shinohara et al. (2010) revealed that the daily consumption of two apples over 2 weeks by human participants lowered the fecal abundance of lecithinase-positive Clostridia, such as *Clostridium perfringens*, a bacterium implicated in food poisoning. Various dietary interventions have shown positive effects on gut microbiota in animal models. For instance, the oral administration of freeze-dried, seedless bitter melon powder to rats decreased the proportion of potential endotoxin-producing opportunistic pathogens, such as *E. coli*, in their fecal microbiota (Bai et al., 2016). Dietary green kiwifruit also lowered fecal *E.* coli abundance (Han et al., 2011), and feeding freeze-dried white mushrooms to mice reduced fecal Clostridia (Varshney et al., 2013). Moreover, broccoli fiber intake specifically reduced the presence of potential pathogens, including *C. perfringens*, *E. coli, and Enterococcus* spp. (Han and Xiao, 2020).

### 3.8 Nuts

Unsaturated fatty acids (PUFAs), fibre, and bioactive substances like phytosterols, polyphenols, and antioxidants (tocopherols) are also abundant in nuts and may have a prebiotic influence on the makeup of the microbiota. Nuts are frequently seen in the Mediterranean diet and plant-based diets (Lamuel-Raventos and Onge, 2017; Yang et al., 2011; Alasalvar et al., 2020). Consuming nuts influences the genus-level microbiota (e.g., while Parabacteroides is decreasing, *Clostridium*, Dialister, Roseburia, and Lachnospira are increasing). The impact of nut eating on gut flora was investigated in a recent thorough review and meta-analysis of randomised controlled trials. However, the specific outcomes differ according to the kind and number of nuts ingested as well as the duration of the intervention (Creedon et al., 2020). A randomized, controlled crossover trial showed that eating 42 g of walnuts daily for 3 weeks increased levels of butyrate-producing bacteria, including Faecalibacterium, Roseburia, *Clostridium*, and Dialister, and other Firmicutes genera (Holscher et al., 2018). However, an 8-week intervention that involved 56.7 g of almonds in young people led to a drop in *B. fragilis* abundance and an increase in α-diversity assessments (Dhillon et al., 2019).

### 3.9 Pulses

Protein from this food group is frequently included in plant-based diets. Additionally, it has high levels of iron, PUFAs and MUFAs, dietary fiber, folate, and certain phytochemicals. Changes in the gut microbiota’s composition and metabolite synthesis have also been linked to pulses’ nutritional value. According to a recent systematic review, consuming pulses can lead to notable changes; however, the effects are not always consistent, particularly in individuals (Marinangeli et al., 2020). For example, eating chickpeas was associated with reduced levels of *Clostridium* cluster XI and I/II and greater levels of *Bifidobacterium* sp. *and Lactobacillus casei/L. bifermentum sp* (Fernando et al., 2010). However, in a population with premetabolic syndrome, pinto beans merely reduced the amount of Eubacterium limosum (Finley et al., 2007). Animal studies revealed more pronounced effects when using pulse flour extracts. Specifically, in mice, black and navy bean flours led to: (1) higher output of short-chain fatty acids (SCFAs); (2) greater populations of Prevotella, S24-7, and *Ruminococcus flavefaciens*; and (3) reduced levels of *Adlercreutzia, Parabacteroides, Streptococcus, Lactococcus, Oscillospira, and Coprococcus* among other bacterial species. The potential pathogen *C. perfringens* was less abundant when navy bean flour was used, whereas black bean flour increased α-diversity. Additional bean-specific alterations were also observed (Monk et al., 2017).

### 3.10 Fermented foods

Sauerkraut, kimchi, kefir, dry-fermented sausage, yoghurt, cheese, kombucha, and miso are examples of fermented foods and drinks that depend on regulated microbial development and frequently include probiotic bacteria (most frequently Leuconostoc, *Streptococcus*, *Lactobacillus*, and Lactococcus) (Dimidi et al., 2019; Tamang et al., 2016). The long-standing tradition of human consumption of yeast and microbial metabolites has seen a recent surge in popularity. This resurgence is driving new research efforts focused on understanding their effects on the host’s gut microbial population and overall health, with particular attention being paid to mental wellbeing (Kim and Shin, 2019; Bourrie et al., 2016). It should not be surprising to learn that consuming “living” fermented foods can alter the intestinal microbial profile by increasing the quantity of microorganisms in the food by 10,000 times (Lang et al., 2014; Marco et al., 2017). For instance, after consuming kimchi, obese women showed marked increases in Bifidobacterium abundance (Han et al., 2015). For example, eating kimchi resulted in significant increases in Bifidobacterium prevalence in overweight women, while fermented soybean milk caused a decline in *C. perfringens* and coliform organisms, and a rise in Bifidobacterium and *Lactobacillus* (Han et al., 2015; Cheng et al., 2005). In a recent investigation on mice, kefir supplementation decreased the quantity of *Bacillus amyloliquefaciens*, *Propionibacterium acnes*, and Lachnospiraceae bacteria 3_1_46FAA. It enhanced the number of Bifidobacterium pseudolongum, Eubacterium plexicaudatum, and L. reuteri. It also changed the gut microbiota’s functional potential in the direction of neuroactive metabolite synthesis (van de wouw et al., 2020). Four weeks of consuming a fermented dairy drink enhanced the activities of the resident microbes. They increased the abundance of a few specific genera, including two unidentified genera of Clostridiales, Gordonibacter, *Lactobacillus*, Holdemania, and an unidentified Mollicutes (RF 9), according to another recent study on humans (Alvarez et al., 2020). According to a recent evaluation of the literature, despite these encouraging findings, there is currently insufficient information to conclude any particular microbial patterns associated with a specific fermented food (Stiemsma et al., 2020). It is challenging to forecast how variations in the microbial makeup of fermented items might explain the disparity. However, more comprehensive clinical research is needed to fully understand how fermented foods affect resident microorganisms and their impact on health (Stiemsma et al., 2020). A recent study examining samples from 115 participants in the American Gut Project revealed a dose-dependent relationship between fermented vegetable consumption and gut microbiome composition (β-diversity). Specifically, individuals who consumed fermented vegetables daily had a gut community that differed from those who consumed them twice a week. Additionally, it was found that the number of bacterial taxa and a microbial functional profile were linked to the consumption of fermented foods, such as *Lactobacillus* species, *Bacteroides*, *Pseudomonas*, Dorea, Prevotella, Oscillospira, and *F. prausnitzii* (Taylor et al., 2020).

### 3.11 Minerals and vitamins

As cofactors in neuronal energy metabolism and neurotransmitter production and metabolism, minerals and vitamins are essential. Gut bacteria produce numerous vitamins, including vitamin K and B vitamins such as folate, riboflavin, and cobalamin (B12) (Hill, 1997; Rowland et al., 2018). Which could be absorbed in part immediately. Since the upper gastrointestinal tract absorbs the majority of vitamins and minerals, typically only trace amounts reach the colon (Sawaya et al., 2012). It is challenging to investigate the impact of these nutrients on the human colonic microbiota, and some contradictory findings have been reported. Nonetheless, there is mounting evidence that the vitamins received by the distal colon can support the microbes that live there as a vital source of nutrition (Waterhouse et al., 2019; Uebanso et al., 2020). Despite conflicting evidence, particularly in human studies, a recent systematic review that pools data from both human and animal research indicates that the gut microbiota’s composition can be influenced by vitamin D levels or supplementation. Key trends are beginning to emerge (Waterhouse et al., 2019). Although there is currently insufficient data to make inferences about the effects of vitamins on particular taxa, it has been proposed that their reciprocal relationship is crucial for preserving both intestinal homeostasis and symbiont abundance. For example, microbiome and vitamins D and A may work in concert to maintain gut barrier integrity and regulate immune activity (Cantorna et al., 2019; Malaguarnera, 2020). The relationship between minerals, vitamins, trace elements, and gut flora is symbiotic: many gut bacteria require specific minerals to thrive. As a result, the delicate balance of the gut microbiome can be affected by both mineral surpluses and deficiencies, potentially favoring the growth of harmful microbes and leading to imbalances (Skrypnik and Suliburska, 2018; Andrews et al., 2003). For example, taking iron supplements increased the number of pathogens, even though other research has demonstrated that extra minerals have either beneficial or neutral impacts on the makeup of the human microbiota like *Clostridium difficile, Staphylococcus aureus, Bacillus cereus, C. perfringens* and *Salmonella* in a cohort of Kenyan children, possibly contributing to gut inflammation (Nitert et al., 2018).

### 3.12 Various polyphenols

Flavonoids (such as flavanones, isoflavones, and anthocyanins) and nonflavonoids (such as Stilbenes, tannins, and lignans) are all members of the phytochemical class known as polyphenols. Fruits, vegetables, chocolate, extra-virgin olive oil, nuts, whole grains, and spices are among the foods rich in polyphenols. Polyphenols are also abundant in beverages, including red wine, green tea, and coffee (Neveu et al., 2010). Intestinal microbes can break down about 90%–95% of polyphenols because they are not absorbed (Duenas et al., 2015). Consuming polyphenols has been linked to several health benefits, including neuroprotective effects, primarily due to their anti-inflammatory and antioxidant properties, enhanced cognitive function in both elderly and young individuals, reduced release of pro-inflammatory cytokines and corticosterone, as well as decreased depressive-like behavior in animal models. (Bastianetto et al., 2015; Gildawie et al., 2018). Higher dietary intake of polyphenols has been linked to a lower prevalence of depression, according to recent observational research (Chang et al., 2016; Godos et al., 2018). Gut bacteria produce numerous vitamins, including vitamin K and B vitamins such as folate, riboflavin, and cobalamin (B12) (n = 82,643 women) (Chang et al., 2016). A recent study on stress in early life in animals also found lower corticosterone levels and improved depression and anxiety-like behavior. Furthermore, the makeup and diversity of microorganisms were altered, especially those associated with the gut-brain axis and microbiota (Donoso et al., 2020).

### 3.13 Various sweetening components

Nowadays, the food industry frequently uses natural (such as stevia) and artificial (like aspartame and saccharin) non-nutritive sweeteners to reduce the amount of sugar in food. Growing research is exploring the impact of sweetener consumption on gut microbiota composition, given diet’s known influence on the microbiota (Plaza-Diaz el al., 2020; Lobach et al., 2019). Even though past research on humans showed negative impacts on the diversity and microbe composition (Frankenfeld et al., 2015; Suez et al., 2014) and Consuming non-nutritive sweeteners was connected in a more recent study to a “dysbiosis”—a term that is becoming less and less useful in microbiome research (Shanahan and Hill, 2019) and a drop in the amount of butyrate (Farup et al., 2019) or Some studies have found that either only a few sweeteners (such as stevia, sucralose, and saccharin) affect the microbial profile (Ruiz-Ojeda et al., 2019) or that it is impossible to prove that sweets have any appreciable impact on the microbiota (Lobach et al., 2019). As a result, precise influence on the microbiota is currently being ascertained and is probably dependent on the individual sweeteners’ chemical characteristics as well as the concentration that enters the colon (Plaza-Diaz et al., 2020). The body processes different sweeteners in distinct ways. Aspartame and saccharin are essentially broken down and absorbed in the upper gastrointestinal tract. In contrast, steviol glycosides (stevia) reach the colon undigested and rely on bacterial fermentation. Sucrose is estimated to enter the colon at a rate of 85%. It has been shown, especially in animal models, that giving sugar (also known as Splenda) causes microbial changes, such as reduced abundance of overall bacteria, decreased *Ruminococcus, Streptococcus, Dehalobacterium, Erysipelotrichaceae*, and *bifidobacteria*, and increased *Proteobacteria, Turicibacteria, Roseburia, Akkermansia, Clostridium symbiosium, Christensenellaceae, Clostridiaceae, and Firmicutes* (Bian et al., 2017; Li Y. et al., 2020; Rodriguez-Palacios et al., 2018). Remarkably, chronic inflammation and glucose intolerance have been connected to several of these microbial changes. Two detrimental health effects are linked to sweetener consumption (Bian et al., 2017). However, short-term sucralose ingestion did not substantially alter the gut microbiota’s composition in small human research, including healthy participants (Thomson et al., 2019). The microbiota’s restricted ability to metabolise sucralose (Magnuson et al., 2016), varying sucralose dosages, and the length of time exposed to sucralose may all be responsible for some of these differences.

### 3.14 Various food-based emulsifiers

Emulsifiers, which include polysorbate-80 (P80), carboxymethylcellulose (CMC), In Western diets. Food additives like carrageenan and arabinogalactan are frequently utilised, which are accustomed to change the flavour of foods and enhance their shelf life, stability, and texture. Animal models have mostly shown that emulsifiers have negative effects on host physiology and the gut microbiota (Jiang et al., 2018) and emulsifier-induced microbial changes have even been proposed to potentially promote pathogen translocation and contribute to inflammatory chronic conditions such as obesity, colon cancer, metabolic syndrome, and gut inflammation (Chassaing et al., 2015; Viennois et al., 2017). Crucially, it has been shown that creatures with extremely little microbiome and germ-free (GF) animals did not experience the same negative health effects when emulsifiers were consumed. This suggests that the negative consequences of emulsifiers on host health may require microbial regulation. Emulsifier consumption in mice has been associated with certain, possibly sex-dependent, microbial alterations, including a decline in the number of *Bacteroides* and a rise in *Helicobacter*, *Salmonella*, *Campylobacter* jejuni, Porphyromonadaceae, and *Clostridium* cluster XI (Yu et al., 2021). The application of CMC resulted in increased presence of Proteobacteria in the community, Burkholderia, and *Clostridium* in female mice. In contrast, P80 treatment in male mice led to higher levels of Veillonella, Burkholderia, *Clostridium*, and *Bacteroides* (Holder et al., 2019).

## 4 Microbiota’s effect on behaviour and brain development

Recently, the microbiota established its role in governing human behaviour combined with brain operations through the bi-directional “microbiome-gut-brain axis (Cryan et al., 2019). Animal studies using GF animals were critical because they proved the brain-gut microbiota relationships through their unusual neurochemical results and behavioural changes (Hoban et al., 2016; Diaz and Heijtz, 2011). Research on animals shows that both the gut microbiota together with correct neurodevelopment impact the development of the hypothalamic-pituitary-adrenal (HPA) axis, which controls stress responses. Researchers face greater difficulties when trying to prove relationships between gut microbiota, microorganisms inhabiting the human digestive tract and brain functions. Modern brain imaging tests showed relationships between the composition of gut microbiota and how the brain functions among patients diagnosed with amnestic moderate cognitive impairment (Liu et al., 2021). Additionally, research has connected the prevalence of particular bacterial species to particular signs and traits of illnesses like autism spectrum disorder (ASD) (Berding and Donovan, 2018).

Additional proof of the microbiota connection can be found in studies conducted on both humans and animals that connect the direct delivery of probiotics, which are helpful microorganisms, to changes in the host’s behaviour and cognition. Research on animals has shown that probiotics (such as B. longum, *Lactobacillus* plantarum, and *Lactobacillus* rhamnosus) can influence cognitive function and have anxiolytic and antidepressant effects (Abildgaard et al., 2017; Ni et al., 2019). Research on animals shows that both the gut microbiota together with correct neurodevelopment impact the development of the hypothalamic-pituitary-adrenal (HPA) axis, which controls stress responses. Researchers face greater difficulties when trying to prove relationships between gut microbiota, microorganisms inhabiting the human digestive tract and brain functions. Modern brain imaging tests showed relationships between the composition of gut microbiota and how the brain functions among patients diagnosed with amnestic moderate cognitive impairment (Marx et al., 2020). For instance, research on the same probiotic strain or prebiotic type is lacking, and effects specific to probiotics and prebiotics are frequently noted.

## 5 One-carbon metabolic (OCM) approaches

**FIGURE 1:**
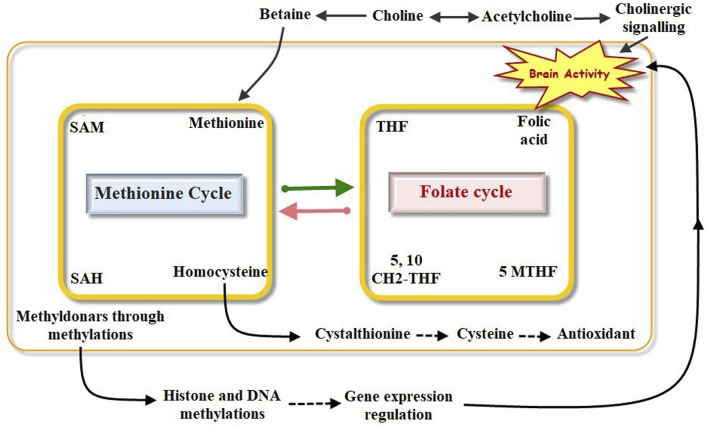
Metabolism of monocarbon.

When methyl-donor micronutrients are present or contribute, several enzymes catalyse chemical reactions that make up one-carbon metabolism. These reactions facilitate a number of processes, including redox state, neurotransmitter synthesis like acetylcholine, nucleotide metabolism, and epigenetic mechanism regulation through SAM formation (Friso et al., 2017; Ducker and Rabinowitz, 2017). Micronutrients that act as methyl donors, specifically Vitamins B6 and B12, betaine, choline, methionine, and folate, are crucial for one-carbon metabolism, a process primarily propelled by the cycles of methionine and folate (Table 3). Changes in micronutrient levels directly affect the processes of epigenetic modification (Figure 1). The key methylation-donor nutrients which sustain one-carbon metabolism operations have been compiled into Table 3. The methionine cycle ultimately produces S-adenosylmethionine (SAM), a vital compound that fuels methylation reactions throughout the cell, including histone and DNA methylation.

**TABLE 3:** The roles that methyl donors play in the metabolism of one carbon.

Methyl donors	Functions	References
Methionine	✓ Antecedent of SAM formations ✓ Preservation of the redox condition ✓ Upkeep of the health of the brain	Kalhan and Marczewski (2012)
Choline	✓ Cholinergic signalling regulation and maintenance ✓ Integrity of the cellular membrane ✓ supporting SAM’s development	Zeisel (1997) Fisher et al. (2002) Tayebati et al. (2015)
Betaine	✓ A methyl donor in the BHMT pathway ✓ Choline precursor ✓ Anti-inflammatory properties	Ueland (2011) Zhao et al. (2018) Slow et al. (2008)
Folic acid	✓ Nucleotide synthesis, normal brain development ✓ Avoiding natural tube defects	Van Goolet al. (2018) Bottiglieri (2013) Mattson and Shea (2003)
V B12	✓ The properties of antioxidants, nucleotide synthesis ✓ Preserving brain health	Froese et al. (2019) Smith et al. (2018)
V B6	✓ Preservation of brain health and the redox state ✓ Participates in the decarboxylation and transamination reactions necessary for the metabolism of several neurotransmitters ✓ Protein metabolism and nucleotide synthesis	Spinneker et al. (2007) Hellmann and Mooney (2010)

### 5.1 Molecular and cellular mechanisms of OCM in brain development

#### 5.1.1 DNA synthesis, repair, and cell proliferation

One-carbon metabolism, particularly through the action of folate, is fundamentally vital for the biosynthesis of DNA. Folate specifically functions as a crucial cofactor for the synthesis of thymidine, which is a key nucleotide component used in DNA replication and repair (Serefidou et al., 2019). Beyond folate, vitamin B12 is also indispensable for *de novo* DNA synthesis and methylation reactions. These processes are critical for the rapid cell division and growth characteristics of the developing fetus and the rapidly expanding brain. A deficiency in folate can have severe consequences for genomic integrity. It can lead to the inappropriate accumulation of uracil in DNA, where it replaces thymidine (Serefidou et al., 2019). This uracil accumulation results in DNA damage and can trigger apoptosis (programmed cell death) of neurocytes, ultimately leading to cognitive dysfunction. This indicates that OCM’s contribution to DNA synthesis is not merely about providing building blocks for growth, but rather about maintaining the quality and viability of neural cells. The integrity of the neural cell genome is a prerequisite for their survival and proper functioning. Animal research has further demonstrated that folic acid (FA) can actively inhibit the apoptosis of astrocytes and delay neurodegeneration, underscoring its protective role beyond mere synthesis. Beyond nucleotides, OCM provides essential one-carbon units (methyl groups) for the synthesis of polyamines, which are also vital for cell proliferation and differentiation (Kalhan and Marczewski, 2012). The importance of OCM in DNA synthesis and cell proliferation is dramatically illustrated by its role in preventing neural tube defects (NTDs). NTDs are severe neurodevelopmental conditions resulting from the failure of neural tube closure during early embryonic development. Folate-mediated OCM is essential for the *de novo* nucleotide biosynthesis required for this critical developmental process. Periconceptional maternal supplementation with folic acid has been shown to reduce the occurrence of NTDs by up to 70% (Kerr et al., 2017). This strong evidence for preventing structural birth defects suggests that OCM’s role in DNA synthesis and repair is critical for the highly coordinated morphogenetic processes during early embryonic neurodevelopment, ensuring the correct formation of brain structures, rather than just supporting undifferentiated cell growth. This indicates a more intricate and specific requirement for OCM in developmental architecture.

#### 5.1.2 Epigenetic regulation (DNA and histone methylation)

One-carbon metabolism plays a pivotal role in epigenetic events, which are recognised as primary mechanisms of developmental programming across various species. S-adenosylmethionine (SAM) is the central molecule is this context, serving as the universal methyl donor utilised by a vast array of DNA, RNA, histone, and protein methyltransferases (Serefidou et al., 2019). SAM is the principal substrate for the methylation of DNA, associated proteins, and RNA, thereby directly influencing gene expression. Methylation, driven by SAM, is an essential cellular process for epigenetic regulation, which profoundly impacts embryonic development and cognitive function. Folate directly influences DNA methylation and repair, primarily through its involvement in nucleotide synthesis (Van der Windt et al., 2021). Notably, hypomethylation of inflammation-related genes, often a consequence of low folate status, may increase the risk of diseases influenced by inflammatory processes. Vitamin B12 is an integral component for facilitating methylation reactions and maintaining genomic stability. Cobalamin (B12) acts as a crucial cofactor for methionine synthase, an enzyme essential for SAM production (Yevgi and Baydar, 2022). Methyltransferases (MTases), a large superfamily of SAM-dependent enzymes, catalyse the transfer of methyl groups from SAM to various atoms (C, O, N, S) in both small-molecule secondary metabolites and macromolecules, including proteins and nucleic acids (Zeisel, 2017). These enzymes are involved in numerous biopathways, including the modification of biopolymers like lipids, proteins, and nucleic acids. The methylation status of DNA and associated histones is highly dependent on the maternal supply of one-carbon metabolites, emphasising the prenatal origins of epigenetic programming. Perturbations in OCM during embryonic development can have a significant “knock-on effect” on the methylation signatures of neural cells within the developing brain, which can subsequently lead to neurodevelopmental aberrations (Kalhan and Marczewski, 2012). DNA methylation patterns exhibit dynamic changes, particularly dramatically during the first 5 years of postnatal life in human neocortical neurons. This observation suggests that while OCM’s epigenetic influence is lifelong, the early postnatal period represents a highly sensitive and dynamic phase for establishing foundational methylation patterns in the brain. Perturbations in OCM during this critical window could have disproportionately significant and potentially irreversible impacts on long-term brain structure, function, and cognitive abilities. SAM-dependent DNA methyltransferases (DNMTs) catalyse the addition of methyl groups to cytosine residues in CpG islands, a process that directly influences neuronal gene expression, synaptic plasticity, and cognitive function (Friso et al., 2017; Ducker and Rabinowitz, 2017). Altered DNA methylation patterns have been specifically linked to neurodevelopmental disorders such as autism and schizophrenia. Histone methylation, another crucial epigenetic modification, controls chromatin accessibility and transcriptional regulation. This process is essential for learning, memory formation, and neuronal differentiation. Dysregulation of histone methylation contributes to psychiatric disorders and neurodegeneration. Specific lysine methylation patterns on histones, such as H3K4, H3K36, and H3K79, are generally associated with active chromatin, while methylation at H3K9, H3K27, and H4K20 typically correlates with inactive or repressed chromatin regions (Dambacher et al., 2010). Human and mouse embryonic stem cells require methionine to maintain the methionine-SAM cycle, which is essential for the formation of H3K4me3, an active histone mark. Specific genes crucial for neuronal differentiation and function have been identified as being impacted by DNA and histone methylation due to disruptions in one-carbon metabolism. For instance, the En2 gene, which is critical for neurogenesis and brain development, has shown specific hypermethylation in contexts of OCM disruption (James et al., 2014). This demonstrates that OCM’s epigenetic influence is not merely a broad “on/off” switch for gene expression but can precisely fine-tune the expression of genes critical for specific neuronal differentiation pathways and brain structural development. Furthermore, the En2 gene in embryonic stem cells is described as being “bivalently marked” by both repressive H3K27me3 and active H3K4me3 histone modifications (Dambacher et al., 2010). The presence of both marks indicates a “poised” state, where a developmental gene is ready for activation upon differentiation. This implies that OCM, by providing methyl groups for these histone modifications, is crucial for maintaining this delicate balance. Disruption of OCM could therefore lead to improper resolution of these bivalent marks, resulting in aberrant or delayed neuronal differentiation and subsequent neurodevelopmental disorders. Other genes like Stum, Tshz3, and Ovol2 have also shown transcriptional dysregulation linked to OCM-mediated histone modifications (Senner et al., 2023).

#### 5.1.3 Myelination and neural integrity

Vitamin B12 is unequivocally essential for comprehensive brain development, including neural myelination, and overall cognitive function. Inadequate vitamin B12 status during critical periods of pregnancy and early childhood has been consistently associated with adverse child health outcomes, particularly impaired cognitive development (Smith et al., 2018) Myelination, the process of forming the myelin sheath, and synaptogenesis (the formation of synapses) are crucial developmental events that commence in the third trimester of gestation and continue to profoundly influence neuronal development during the first few years of life. Myelin itself is a fatty substance that ensheaths nerve fibres, providing electrical insulation and significantly facilitating the rapid and efficient transmission of nerve impulses. It dramatically increases the speed of an action potential by 10–100 times compared to an unmyelinated axon. The integrity of myelination is directly linked to rapid information transfer, which is essential for optimal cognitive functioning, as well as emotional and behavioural regulation and decision making (Khelfaoui et al., 2024). This process of myelination continues for decades in the human brain, highlighting its ongoing importance beyond early childhood. A deficiency in vitamin B12 can lead to the degradation of the myelin sheath, directly impairing nerve signal transmission. This manifests clinically in various neurological symptoms, including numbness, tingling, and muscle weakness. S-adenosylmethionine (SAM) dependent methylation reactions are crucial for myelin production. When SAM production is reduced or methylation reactions are impaired due to conditions like B12 deficiency, it leads to diminished methylation capacity and subsequent damage to myelin (Friso et al., 2017). Consequently, B12 deficiency can result in defective myelin synthesis and demyelination, particularly evident in the spinal cord, manifesting as subacute combined degeneration (SCDC). The concept of “adaptive myelination” reveals that myelination is not a static developmental event. More active brain circuits gain more myelin, a process essential for learning, attention, and memory. This adaptive myelination is plastic and responsive to experience, continuing to influence neural circuits throughout life. This suggests that OCM’s role in myelin synthesis (via SAM) is not limited to the initial formation but is crucial for the brain’s ongoing ability to optimize neural circuits in response to experience throughout life. This significantly broadens OCM’s relevance from static development to dynamic, lifelong cognitive function and neural plasticity (Miranti et al., 2017). Furthermore, beyond general cognitive impairment, the research links impaired myelination due to OCM dysfunction to specific severe conditions such as subacute combined degeneration and even implicates adaptive myelination in the progression of addiction and epileptic seizures. This suggests that the integrity of myelin, which is heavily reliant on OCM, is a critical and sensitive vulnerability point for a broad spectrum of neurological dysfunctions, extending OCM’s impact beyond just cognitive decline to encompass broader neurological health and disease.

#### 5.1.4 Neurotransmitter synthesis and function

Folate is an essential nutrient for the biosynthesis of crucial neurotransmitters that regulate mood, stress responses, motivation, and cognitive performance. These include serotonin (5-HT), noradrenaline (NA), and dopamine (DA). Vitamin B12, through its integral role in S-adenosylmethionine (SAM) production, serves as an important methyl donor for neurotransmitter synthesis (Friso et al., 2017). Specifically, B12 acts as a cofactor in the production of several key neurotransmitters, including serotonin and dopamine. Pyridoxal phosphate, the active form of Vitamin B6, is also required for various steps in neurotransmitter synthesis pathways. Choline, while not a B vitamin, is a precursor to the neurotransmitter acetylcholine and plays a significant role in its synthesis. Impaired methylation reactions, often resulting from low SAM levels due to OCM dysfunction, can directly disrupt serotonin signalling, thereby contributing to the development of depression and anxiety disorders (Miranti et al., 2017). Low folate status has been directly associated with depression, a link potentially mediated by elevated homocysteine (Hcy) levels, as Hcy is toxic to the dopaminergic system. This specific targeting of a key neurotransmitter system offers a direct causal link between OCM imbalance and specific neurological pathologies, such as depression (which is strongly linked to dopamine dysregulation) (Kim et al., 2018). This moves beyond general toxicity to a more refined understanding of the pathological pathways. Genetic variations, such as mutations in the MTHFR gene, a key enzyme in folate metabolism, can significantly affect folate metabolism. This can lead to abnormal levels of key neurotransmitters like serotonin and dopamine, contributing to psychiatric conditions such as depression, anxiety, ADHD, and autism spectrum disorders. SAM, being crucial for their creation, is directly impacted by MTHFR mutations (El-Khawaga et al., 2024). The explicit connection between OCM and its B vitamin cofactors not just to general brain function but to the synthesis of specific neurotransmitters and, crucially, to the etiology of various mental health and neurodevelopmental conditions like depression, anxiety, ADHD, and schizophrenia indicates that OCM is a fundamental regulator of the brain’s neurochemical environment, directly impacting mood, motivation, and cognitive control (Duncan et al., 2025). This is a deeper implication for mental health beyond just broad “brain development”.

#### 5.1.5 Neurotrophin production

Research strongly suggests that folate may exert a beneficial influence on the production and activity of neurotrophins, including brain-derived neurotrophic factor (BDNF) and nerve growth factor (NGF) (Zou et al., 2024). BDNF is a critical neurotrophin for maintaining brain homeostasis and promoting neurogenesis. It plays a pivotal role in regulating neural circuit development, fostering neuronal growth, and enhancing synaptic plasticity. NGF, among its various functions, is essential for the survival of cholinergic neurons within the central nervous system (CNS) and actively promotes the growth and repair of nerve cells. Disturbingly, low maternal folate levels, as well as imbalance between folate and vitamin B12, have been directly associated with reduced levels of both BDNF and NGF (Ducker and Rabinowitz, 2017). Low neurotrophin levels are further suggested to increase the risk of preeclampsia in the mother, a condition that, in turn, has been shown to elevate the risk of neurodevelopmental disorders in the offspring, such as ADHD, epilepsy, lower cognitive ability, and greater cognitive decline in later life. This reveals a complex, indirect causal chain, indicating that maternal OCM status can influence fetal brain development not only through direct nutrient supply but also by impacting maternal physiological conditions that, in turn, affect the fetal neurological environment (Duncan et al., 2025). Animal research has provided evidence that folic acid supplementation can effectively increase serum levels of BDNF (Rezavanimehr et al., 2024). The connection between OCM and neurotrophins suggests that OCM’s importance extends beyond merely supporting initial brain formation. Neurotrophins are crucial for neuronal growth, survival, and synaptic plasticity (Tsimpolis et al., 2024). The explicit role of B12 in NGF production for “neural regeneration” and repairing damaged nerve tissues implies that OCM is crucial for the brain’s ongoing capacity for adaptation, resilience, and even repair throughout life, not just during early developmental stages. This has a more profound and long-term implication for brain health.

## 6 Stress-related disorders, epigenetic modifications, and dietary sources of methyl donors

Scientific studies have demonstrated that early-life experiences significantly influence the human stress response mechanism and the development of the hypothalamic-pituitary-adrenal (HPA) axis (Koe et al., 2014). Distinct research gaps emerge regarding how specific methyl donors (e.g., choline, folic acid, methionine, vitamins B6 and B12) influence the epigenetic regulation of genes implicated in stress-related disorders during critical periods of brain development. Childhood experiences can alter the programming of the stress response system, potentially through epigenetic modifications, such as DNA methylation. These modifications can alter the expression of stress-related genes, resulting in lasting changes in brain function and behavior (Saunderson et al., 2016; Babenko et al., 2015). Early-life methyl-donor micronutrient supplementation or deficiency that affects the development of children’s brains may impact their behavior in the long run. The significance of methylation and how early life experiences influence the development of the stress axis will be discussed in this section. The following sections analyze the intricate interplay of methylation changes, micronutrients, and stress-related/neurodevelopmental disorders.

### 6.1 Exploring the effects of dietary donors on neuroprotection - a holistic overview

The causes of numerous diseases are multifaceted, involving both inherited factors and environmental exposures, with epigenetic regulation of genes playing a key role. Dietary micronutrients providing methyl groups, which fuel one-carbon metabolism pathways, are indispensable for brain development and its subsequent function through their involvement in S-adenosylmethionine (SAM)-dependent methylation. Folate, vitamin B12, choline, and betaine are essential examples of such methyl donors, impacting neurodevelopmental processes and vulnerability to psychiatric conditions (Bekdash, 2023). Maternal folate consumption is a well-established protective factor against neural tube defects and is also related to positive cognitive development in children. Research indicates that adequate consumption of Vitamin B12 and Folate during the early phases of pregnancy by the mother promotes cognitive performance and language skills in children. Improved academic performance among teenagers has been associated with higher dietary folate levels. These results are supported by research on animals. While a vitamin B deficiency raises homocysteine levels, causing neuronal damage and cognitive deficits, prenatal folate deficiency alters brain development by decreasing the number of progenitor cells in the fetal neocortex. Methyl-donor supplementation can correct the depressive-like behaviour and altered lipid metabolism shown in rats with depression models generated by maternal separation (Paternain et al., 2016). Additionally, methyl donors have been shown to lessen anxiety-like behaviors and oxidative damage in rats under chronic stress, confirming their potential as nutri-therapeutic agents. Dysregulation of methyl donors is implicated in diseases like Alzheimer’s disease (AD), while S-adenosyl methionine (SAM) regulates diverse neural functions. Amyloid beta plaque buildup and tau hyperphosphorylation are hallmarks of AD, and pathogenesis is aided by dysregulated one-carbon metabolism (Bekdash, 2023). High levels of homocysteine and low levels of vitamin B are linked to cognitive deterioration. Insufficiency is associated with hypomethylation of the presenilin 1 (PSEN1) gene, which increases the synthesis of amyloid beta. By lowering tau phosphorylation and the load of amyloid plaque, SAM supplementation counteracts these effects. By raising the DNA methylation of the PSEN1 and APP genes, folate alterations in AD models also lower amyloid beta levels. Choline, vital for neurotransmission, membrane structure, and the creation of betaine and acetylcholine, offers neuroprotective benefits. Various crucial micronutrients and their crucial mechanistic role are depicted in Supplementary Table S1. Supplementation with choline in Alzheimer’s Disease (AD) mouse models has demonstrated improvements in spatial memory, reduced amyloid beta plaques, and decreased neuroinflammation through the reduction of microglial activation (Velazquez et al., 2019). While current AD treatments rely on acetylcholinesterase inhibitors to boost acetylcholine levels, their effectiveness is limited. Notably, choline supplementation during the perinatal period enhances hippocampal function, reduces amyloid burden, and improves cholinergic signaling, underscoring its crucial role in early brain development. Furthermore, sustained choline intake in AD models has shown promise in restoring neurotransmitter balance, improving synaptic function, and alleviating anxiety alongside memory impairments.

## 7 Mechanisms of associative epigenetics and methyl-donor micronutrients

The science of epigenetics focuses on evaluating modifications that do not alter the basic DNA sequence. Conrad Waddington first proposed the field of epigenetics in 1942. It is concerned with how experiences and the environment affect gene activity through procedures including DNA methylation and histone alterations. Enzymes referred to as “writers” and “erasers,” which alter epigenetic marks, control these alterations, making the process reversible and hence a possible target for therapeutic interventions. Moreover, metabolites affect the activity of these enzymes, establishing a connection between epigenetic control and metabolic alterations. A critical aspect of epigenetic changes is the one-carbon metabolic process, which includes the cycles of methionine and folate. S-adenosylmethionine (SAM) is crucial for histone methylation and DNA, acting as the primary methyl donor, and is produced by this system. SAM then regulates gene expression, influences neurodevelopment, and impacts several other biological processes (Serefidou et al., 2019). S-adenosylmethionine (SAM) synthesis, crucial for brain health, relies on a network of micronutrients involved in one-carbon metabolism. Multiple methyl donors, including choline, betaine, methionine, and folate, as well as vitamins B6 and B12, combine to modify DNA and histone methylation patterns (Bekdash, 2023). Furthermore, one-carbon metabolism is crucial for producing neurotransmitters and other essential cellular components, both of which are vital for maintaining proper brain function. Prenatal, postnatal, and adolescent periods are among the early developmental stages that are particularly susceptible to environmental factors, including stress, nutrition, and pollutants, which can have long-term health and neurodevelopmental effects. Methyl-donor micronutrients are crucial during these phases, as deficiencies can lead to behavioral abnormalities, cognitive decline, and neurological issues. Methyl-donor deficiency has been linked in studies to negative neurodevelopmental consequences. For instance, mice lacking choline showed reduced global methylation in the hippocampus, which affected cell cycle-related genes and ultimately affected brain development. Maternal intake of methyl donors, including choline and folate, can alleviate developmental problems in offspring (McKee and Reyes, 2018). Choline supplementation, for instance, enhanced the expression of genes linked to anxiety and cognitive issues in the adult progeny of pregnant rats lacking iron. Likewise, folate supplementation late in pregnancy reversed developmental defects by restoring normal levels of microRNAs, which are critical for embryonic development and brain function (Geoffroy et al., 2017). Dietary methyl donors also affect the brain function of adult offspring. In mice given a diet high in fat, methyl donor supplementation resulted in overall reduced DNA methylation in reward-related brain regions, such as the nucleus accumbens and prefrontal cortex, accompanied by altered behavior and metabolic rate. Regarding mental health, epigenetic changes are implicated in anxiety and depression. Studies in rats revealed that high-anxiety individuals displayed changed amygdala patterns of DNA methylation, especially in the genes that are related in mood regulation and synaptic activity. Increasing dietary methyl-donor intake in these animals improved anxiety and depressive behaviors, highlighting the epigenetic influence on mental wellbeing.

### 7.1 Functional approaches of methyl-donor micronutrients and its allied epigenetic processes in mental and metabolic conditions

During crucial developmental periods, a mother’s consumption of methyl-donor micronutrients has a significant impact on how metabolic genes are epigenetically regulated in her offspring, with substantial consequences for both metabolic and mental wellbeing. Folate, choline, and vitamin B12 are essential nutrients involved in one-carbon metabolism, a crucial process that encompasses both the production of neurotransmitters and DNA methylation, both of which are vital for brain development (Schaevitz et al., 2014). Disturbances in these processes throughout the development of the embryo can result in “fetal programming,” indicating that early nutritional experiences can reshape a person’s long-term health, affecting their vulnerability to obesity, metabolic illnesses, and mental health problems. Mental health illnesses are directly associated with obesity, a global public health concern. By altering the epigenetic mechanisms that govern gene expression in the brain, environmental variables, including maternal nutrition, may play a role in the non-genetic causes of metabolic diseases, such as obesity (Lavallee et al., 2021). Deficiency in methyl-donor nutrients, like vitamin B12 and folate, in mothers during pregnancy influences the offspring’s brain’s expression of key metabolic genes, resulting in abnormalities in energy balance and appetite regulation, according to several rodent studies. These epigenetic modifications can have a lasting impact on mental health and metabolism. Micronutrients that donate methyl groups are crucial for brain development and act as protectors of the nervous system by influencing gene expression through DNA and histone methylation. Insufficient or unbalanced levels of these micronutrients during pregnancy or early life can disrupt the brain’s epigenome, potentially causing neurodevelopmental and metabolic problems (Zeisel, 2009). For instance, it has been demonstrated that choline supplementation during pregnancy alters the hypothalamic circuits that control an offspring’s appetite, and that a mother’s folate deficiency is linked to modifications in the expression of genes involved in energy management. It was also found that a mother’s consumption of methyl donors can positively impact a child’s cognitive and behavioral development, underscoring the significance of sufficient nutrient intake during key stages of development. There is a complicated and multifaceted interaction between mental health issues, brain health, and nutrition. Nonetheless, research indicates that the type and amount of nutrients ingested in early development have a significant influence, how the brain develops and the probability of mental health issues later in life. For example, obesity, mental health issues, and insulin resistance in children have all been linked to vitamin B12 and folate shortages during pregnancy. Similarly, it has been shown that maternal choline intake affects the development of brain inhibition and the likelihood that children may experience diseases such as anxiety and schizophrenia.

## 8 Derived product as vitamin B complex

Vitamin B12, readily available in animal products (meat, fish, dairy), is essential for one-carbon metabolism, a process critical for gene methylation. When B12 levels are low, SAM depletion results, disrupting DNA and histone methylation, which in turn negatively affects gene expression and neuronal function (Lobos and Regulska-Ilow, 2021). Given that maternal deficiencies have been connected to depression and oxidative stress neuropathy in both the mother and her unborn child, this is especially concerning during pregnancy (Socha et al., 2024). Folate, vitamin B6, and vitamin B12 help regulate neurotransmitter production, a process essential for mood regulation (Cicero and Minervino, 2022). By restoring normal homocysteine levels and enhancing the synthesis of serotonin, norepinephrine, and dopamine—neurotransmitters linked to mood disorders—there is proof that taking Vitamin B12 supplements can lessen depressive symptoms. The significance of these micronutrients in mental health is further highlighted by long-term research that indicates adults who consume more B12 and B6 Vitamins are less likely to have depressed symptoms.

### 8.1 Folate in brain function and cognitive health

Folate is necessary for nucleotide synthesis and brain development. A deficiency in folate during pregnancy, especially during the crucial period of gestational days 11–17 (GD11-GD17), can disrupt the differentiation of neuronal stem cells and increase neuronal apoptosis within the developing fetal brain (da Silva et al., 2025); however, folate supplementation has been shown to counteract these adverse effects. Furthermore, folate influences metabolic genes, as evidenced by rodent studies showing that maternal folate deficiency leads to altered hypothalamic expression of metabolic genes, specifically increased expression of Pomc (pro-opiomelanocortin) – a critical gene regulating appetite–along with changes in leptin and insulin levels, which impact energy homeostasis (Jiao et al., 2025). Moreover, maternal folate Supplementation has proven to be effective in alleviating the consequences of early-life stress, as observed in rat models where folic acid reversed autistic-like and anxiety behaviors, likely through modulation of DNA methylation patterns in genes like GFAP (glial fibrillary acidic protein) and BDNF (brain-derived neurotrophic factor) both of which are integral to brain development and neuronal support. Research involving pregnant women suggests that improper distribution of vitamin B12 and folate may contribute to obesity (González-Ludlow et al., 2025), as well as the development of insulin resistance in their newborn children. Equitable health and metabolic wellness depend directly on sufficient micronutrient consumption during pregnancy.

### 8.2 Choline in brain function and cognitive health

Foods like meat, fish, and eggs contain choline, which is vital for brain development, especially during the crucial prenatal stages. It aids in the production of phospholipids like phosphatidylcholine and the neurotransmitter acetylcholine, both of which are essential for preserving the cellular membranes’ integrity. Because it contributes to methylation processes through the synthesis of SAM (S-adenosylmethionine), choline’s function goes beyond neurotransmission (Yan et al., 2025). Choline deficit during pregnancy in rodents affects progenitor cell migration and proliferation, which hinders the formation of the embryonic hippocampus and may result in long-term brain disorders. Conversely, choline supplementation in the mother’s diet can change how neuropeptides are expressed in the brain, thereby impacting food intake and energy balance. Research also indicates a potential connection between schizophrenia development and maternal choline consumption, as choline may help to counteract the risk associated with impaired cholinergic neurotransmission, particularly within the alpha-7 nicotinic receptor pathway (King and Plakke, 2025). Human studies corroborate these findings, demonstrating that lower maternal plasma choline levels correlate with developmental delays and sensory processing deficits in infants, reinforcing choline’s importance for healthy neurodevelopment.

## 9 Derived product as methionine and SAM

S-Adenosylmethionine (SAM), generated from methionine, acts as a primary methyl donor in crucial biological processes like DNA methylation, neurotransmitter synthesis, and phospholipid synthesis (González-Ludlow et al., 2025). Insufficient methionine impairs SAM production, resulting in deficient methylation patterns and altered gene expression, potentially predisposing individuals to neuropsychiatric illnesses. Notably, elevated homocysteine, an indicator of decreased SAM and vitamin B12, has been found in individuals with depression and schizophrenia, highlighting the critical link between one-carbon metabolism and mental wellbeing. Methionine supplementation has been demonstrated to normalise stress responses and reverse epigenetic alterations brought on by early-life stress in animal models. With some encouraging findings, clinical research has also looked into the use of SAM supplements to treat major depressive disorder (MDD) (Yan et al., 2025). Aberrant gene methylation has been connected to decreased SAM activity and dysregulation of the methionine cycle in schizophrenia cases, which may aid in the onset of the illness.

## 10 Mechanistic approaches of iron micronutrients catalysis and effect on brain development and cognitive health

**FIGURE 2:**
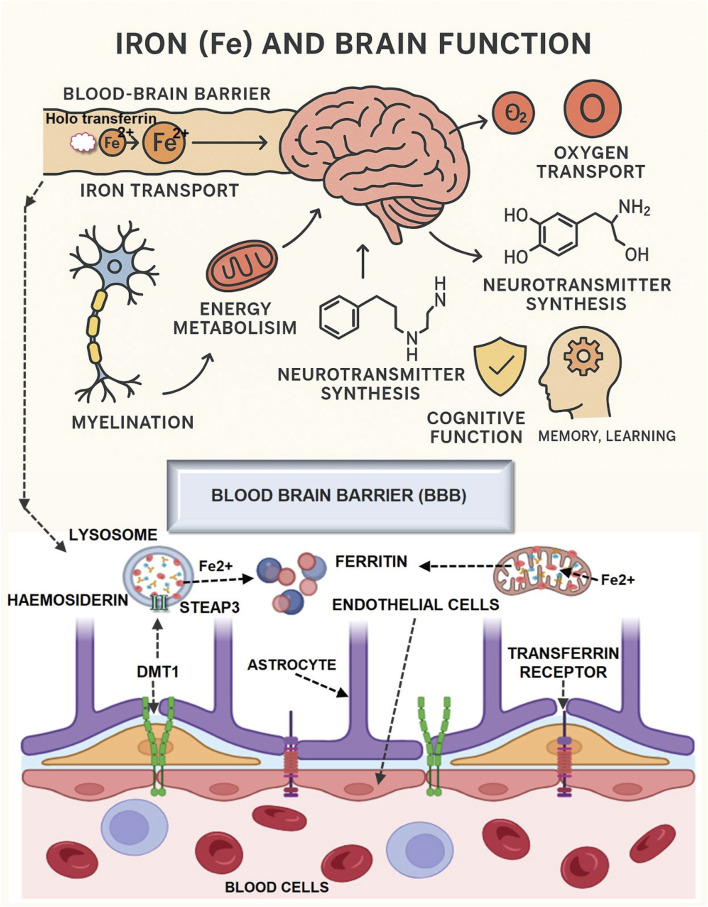
Mechanism of Iron micronutrient on brain development and function.

Iron metabolism in the brain is tightly regulated through specialised mechanisms at the blood-brain barrier (BBB) and cellular pathways to balance iron’s essential roles in neuronal function with its potential toxicity (Figure 2) (Kim et al., 2025). Iron is a key component of haemoglobin and cytochromes, enabling oxygen transport and mitochondrial respiration in neurons (Catapano et al., 2025). This supports the high metabolic demands of brain cells, especially during development and active cognitive processes. Here’s how acute iron metabolism supports brain function:

### 10.1 Iron uptake at the blood-brain barrier

Iron enters the brain mainly via transferrin-mediated uptake across the blood-brain barrier (BBB) (Kim et al., 2025). Within the brain, iron is distributed to neurons and glial cells through tightly regulated mechanisms involving transporters like DMT-1, ferroportin, and regulatory hormones such as hepcidin, which help prevent both deficiency and toxicity (Szymulewska-Konopko et al., 2025).

#### 10.1.1 Transferrin-mediated transport

Circulating iron (Fe^3+^) binds to transferrin (TF), forming a TF-Fe^3+^ complex that interacts with transferrin receptors (TfR1) on brain microvascular endothelial cells (BMVECs) (Singh M. A. F. et al., 2014). This complex undergoes receptor-mediated endocytosis.

#### 10.1.2 Acidic endosomal release

Within endosomes, H^+^-ATPase lowers the pH (∼5.5–6.5), reducing Fe^3+^ to Fe^2+^ via STEAP proteins (Ju and Hang, 2025). Fe^2+^ is transported into the cytosol by divalent metal transporter 1 (DMT1) (Singh N. et al., 2014; Ju and Hang, 2025).

#### 10.1.3 Alternative pathways

Non-transferrin-bound iron (NTBI) enters via ZIP14/Slc39a14 or calcium channels in iron-overload conditions (Singh N. et al., 2014; Ju and Hang, 2025).

### 10.2 Iron export to brain parenchyma

#### 10.2.1 Ferroportin-mediated efflux

Fe^2+^ is exported across the abluminal membrane of BMVECs via ferroportin (Fpn), the sole iron efflux protein (Singh N. et al., 2014; Ju and Hang, 2025).

#### 10.2.2 Oxidation by ceruloplasmin

Astrocytic ceruloplasmin (Cp) oxidises Fe^2+^ to Fe^3+^, enabling binding to brain transferrin (secreted by choroid plexus and oligodendrocytes) or citrate/ATP for distribution (Singh N. et al., 2014; Ju and Hang, 2025).

### 10.3 Cellular iron utilization and storage

#### 10.3.1 Neurotransmitter synthesis and regulation

Iron acts as a cofactor for enzymes involved in synthesising neurotransmitters such as dopamine, serotonin, and norepinephrine. For example, tyrosine hydroxylase (for dopamine) and tryptophan hydroxylase (for serotonin) require iron, directly linking iron status to cognitive and emotional regulation.

#### 10.3.2 Neuronal and glial uptake

Neurons and oligodendrocytes acquire TF-bound Fe^3+^ via TfR1, while astrocytes take up NTBI via DMT1 (Singh N. et al., 2014; Ju and Hang, 2025).

#### 10.3.3 Ferritin storage

Excess iron is sequestered in ferritin nanocages (composed of H- and L-subunits) to prevent Fenton reactions (Hare et al., 2013; Ju and Hang, 2025).

#### 10.3.4 Mobilisation via ferritinophagy

Lysosomal degradation of ferritin releases Fe^2+^ during cellular demand (Ju and Hang, 2025).

### 10.4 Myelination and synaptic development

Iron is essential for oligodendrocytes to produce myelin, the insulating sheath around nerve fibres that accelerates electrical signaling (McCann et al., 2020). Iron deficiency can impair myelination, slowing neural transmission and affecting learning and memory (Ferreira et al., 2019).

### 10.5 Neurogenesis and synaptogenesis

Adequate iron supports the generation of new neurons and the formation of synapses, processes fundamental for brain plasticity, learning, and memory (Zachariou et al., 2024).

### 10.6 Key regulatory proteins

Various key regulatory proteins, like Hepcidin, modulate ferroportin activity by inducing its internalisation in response to systemic iron levels (Ju and Hang, 2025). STEAP/DMT1 facilitate the reduction and transport of iron within endothelial cells (Ju and Hang, 2025). Cp/Hp Oxidize Fe^2+^ to Fe^3+^ for safe binding to TF or storage (Singh N. et al., 2014; Ju and Hang, 2025).

### 10.7 Consequences of dysregulation

Acute iron overload disrupts redox balance, generating hydroxyl radicals (·OH) via Fenton reactions (Fe2++H2O2→Fe3++OH−+⋅OH) (Ju and Hang, 2025). This causes lipid peroxidation, DNA damage, and protein oxidation, exacerbating neuroinflammation and neuronal death (Hare et al., 2013; Ju and Hang, 2025). The brain’s iron metabolism relies on precise coordination between BMVECs, astrocytes, and neurons to maintain homeostasis, ensuring adequate supply for neurotransmitter synthesis, myelination, and mitochondrial function while minimising oxidative stress (Singh N. et al., 2014; Hare et al., 2013; Ju and Hang, 2025).

## 11 Zinc is essential for brain development through several tightly regulated mechanistic pathways

### 11.1 Transport and cellular homeostasis

**FIGURE 3:**
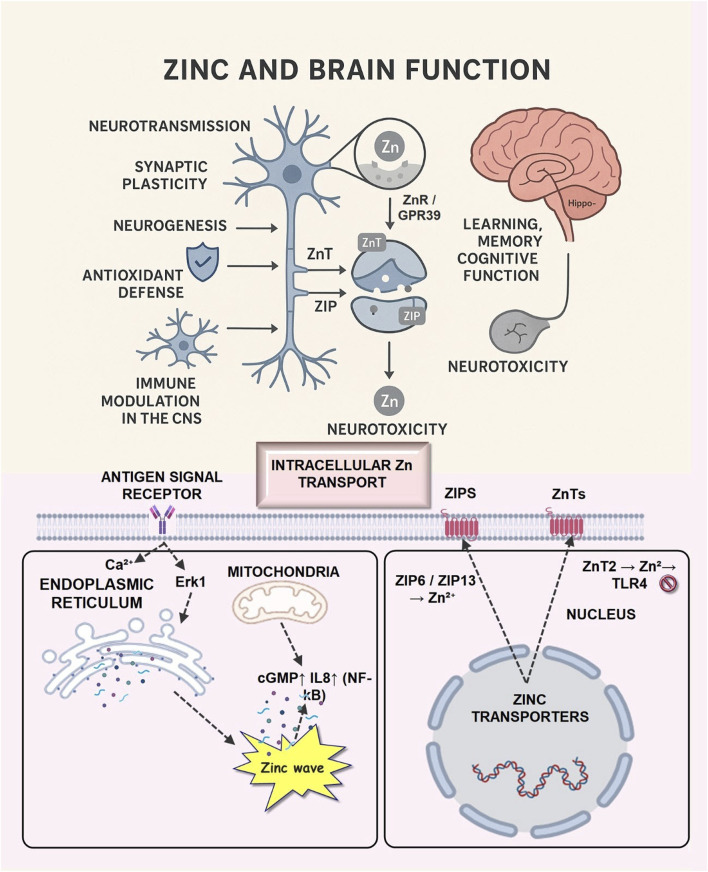
Mechanism of Zinc micronutrient on brain development and function.

Zinc cannot freely diffuse across cell membranes; it requires specialised transporters such as ZIPs (Zrt-Irt-like proteins), ZnTs (zinc transporter family), and metallothioneins (MTs) to enter and move within brain cells (Figure 3) (Li et al., 2022; Willekens and Runnels, 2022; Chamakioti et al., 2024). MTs act as zinc reservoirs, releasing or binding zinc in response to cellular redox changes, thereby maintaining zinc homeostasis during critical periods of brain growth (Chamakioti et al., 2024).

### 11.2 Neurogenesis and cell differentiation

Zinc finger proteins (ZFPs) are crucial transcription factors that regulate gene expression during neurogenesis, affecting stem cell proliferation, neuronal differentiation, and cell survival (Chamakioti et al., 2024). Zinc influences signalling pathways such as STAT1/STAT3 and TGFβ, which are vital for embryonic CNS development and neuronal differentiation (Chen et al., 2024; Chamakioti et al., 2024). Zinc deficiency disrupts these pathways, leading to impaired cortical structure, abnormal neurogenesis, and cognitive deficits (Chamakioti et al., 2024).

### 11.3 Synaptic transmission and plasticity

Zinc is stored in synaptic vesicles (via ZnT3 transporter) and is co-released with glutamate at excitatory synapses, especially in the hippocampus—a region critical for learning and memory (Li et al., 2022; Chen et al., 2024; Chamakioti et al., 2024). Upon synaptic activity, vesicular zinc modulates neurotransmission by interacting with postsynaptic receptors (e.g., NMDA, AMPA), influencing synaptic plasticity, learning, and memory formation (Li et al., 2022; Chen et al., 2024; Chamakioti et al., 2024). Zinc also acts as a neuromodulator, refining sensory processing and supporting both short-term and long-term synaptic plasticity (Chen et al., 2024; Chamakioti et al., 2024).

### 11.4 Antioxidant defence and cellular protection

Zinc, through MTs, helps scavenge reactive oxygen species (ROS) and prevents neuronal toxicity, which is particularly important during rapid brain growth and in protecting developing neurons from oxidative stress (Chamakioti et al., 2024). Zinc deficiency increases ROS production, impairs mitochondrial function, and inhibits MT activity, leading to compromised neuronal survival (Takeda, 2000; Chamakioti et al., 2024).

### 11.5 Regulation of hormones and stress response

Zinc modulates glucocorticoid hormone levels, which, if dysregulated due to zinc deficiency, can lead to excitotoxicity, impaired neurogenesis, and increased neuronal death in developing brain regions (Chamakioti et al., 2024).

### 11.6 Influence on signal transduction

Zinc modulates various intracellular signalling cascades (such as ERK1/2 and p53), which are essential for cell cycle regulation, apoptosis, and neuronal precursor proliferation (Chamakioti et al., 2024).

Zinc’s multifaceted roles in brain development are mediated by its involvement in transport, gene regulation, neurotransmission, antioxidant defense, and hormonal modulation, making it indispensable for proper neural development and function (Li et al., 2022; Chen et al., 2024; Willekens and Runnels, 2022; Chamakioti et al., 2024).

## 12 Mechanistic role of Omega-3 fatty acids in brain function and development

**FIGURE 4:**
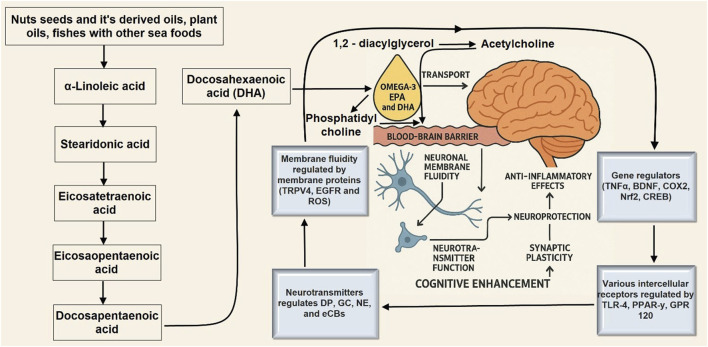
Mechanism of Omega-3 micronutrient on brain development and function.

Omega-3 fatty acids, particularly docosahexaenoic acid (DHA) and eicosapentaenoic acid (EPA), are essential polyunsaturated fatty acids that play crucial roles in brain structure and function (Figure 4) (Tahir and Siddiq, 2025).

### 12.1 Membrane structure and fluidity

DHA is a major structural component of neuronal cell membranes, especially in the grey matter, accounting for over 40% of total omega-3 PUFAs in neuronal tissue (Lauritzen et al., 2016). DHA modulates key biophysical properties of membranes, such as fluidity, acyl chain order, and the formation of lipid rafts, which are crucial for cell signaling and synaptic function (Dighriri et al., 2022). Enhanced membrane fluidity supports efficient neurotransmitter release and signal transmission between neurons.

### 12.2 Neurogenesis and cell differentiation

DHA promotes the differentiation of neural stem cells into neurons, supporting neuronal growth and development (Lauritzen et al., 2016). Both DHA and EPA influence transcription factors that regulate neural cell fate, with DHA favoring differentiation and EPA affecting proliferation (Dyall, 2015).

### 12.3 Neuroprotection and anti-inflammatory effects

Omega-3 fatty acids exhibit neuroprotective properties by reducing neuronal apoptosis (cell death), mitigating oxidative stress, and lowering inflammation in the brain (von Schacky, 2021). They upregulate antiapoptotic proteins and downregulate pro-apoptotic proteins, contributing to neuronal preservation and resistance to neurodegenerative processes (von Schacky, 2021).

### 12.4 Cerebral blood flow

EPA and DHA derivatives act as vasoactive molecules, improving cerebral blood flow and reducing the risk of vascular events like stroke, which is vital for maintaining brain health (von Schacky, 2021).

### 12.5 Role in brain development

#### 12.5.1 Critical periods

DHA accumulates rapidly in the fetal brain during the last trimester of pregnancy and continues at high rates through the first 2 years of life, a period of intense brain growth and development (Lauritzen et al., 2016). Endogenous synthesis of DHA is limited, making dietary intake (e.g., from breast milk or formula) essential for infants and young children.

#### 12.5.2 Cognitive and sensory development

Adequate omega-3 intake, especially DHA, is linked to optimal development of sensory, perceptual, cognitive, and motor neural systems in early life (Shahabi et al., 2025). DHA-rich regions of the brain are associated with functions such as planning, problem-solving, attention, and memory. Studies indicate that low DHA levels are associated with learning and memory deficits, while adequate levels support normal IQ and cognitive performance.

#### 12.5.3 Long-term brain health

Omega-3 fatty acids continue to support brain structure and function throughout life, with evidence suggesting roles in preventing cognitive decline and supporting mental health in adulthood. Omega-3 fatty acids, especially DHA, are indispensable for building and maintaining neuronal membranes, supporting neurogenesis, protecting against neurodegeneration, and ensuring optimal brain development and function from the fetal stage through adulthood (Shahabi et al., 2025).

## 13 An incite of micronutrient deficiency and allied consequences

Micronutrient deficiency symptoms typically emerge only after prolonged depletion. The symptoms related to nutrient deficiencies can be varied and often resemble other conditions. Common signs include recurring infections, skin issues, and anaemia, which may be microcytic (due to deficiencies in iron or copper) or macrocytic (due to vitamin B12 deficiency) (González-Ludlow et al., 2025). Neuropathy and loss of appetite (anorexia) can also occur. Deficiency diseases of specific severity will manifest when individuals lack proper amounts of vitamins C, D, A, or K, together with niacin. These diseases include scurvy, osteomalacia, pellagra, hemorrhagic disorders, and night blindness. The body can show acute symptoms from chronic deficits when thiamine deficiency happens after nutrient substrate administration or through lactic acidosis (Attaluri et al., 2018). Furthermore, leaking from capillaries, driven by cytokine release during inflammatory responses (infectious or non-infectious), can lead to the redistribution of the binding proteins of micronutrients between compartments. After that, all micronutrient’ plasma levels drop. Redistribution processes also contribute to the decline because the area closest to the location of inflammation has the largest concentration of micronutrients with antioxidant properties. A further contributing factor to the decline is the escalating demand for micronutrients, which are essential as cofactors in intermediate metabolism and as antioxidants. As their condition worsens, their consumption rises. Recognising the possible increased loss of micronutrients in burn patients, as well as in urine, drainages, and repeated haemodialysis, is crucial (Friedrich et al., 2025). Hepcidin synthesis regulates a number of intricate processes that lead to the sharp decline in iron plasma levels. The drop in plasma concentrations of micronutrients is more of a hypothesis than a verified result. Only a few micronutrients can have their amounts regularly measured. The micronutrient problem raises a number of enquiries.

### 13.1 Motor neuron disease, including amyotrophic lateral sclerosis

Micronutrient supplementation in neurology serves multiple purposes. One is preventing deficiencies that can cause nutritional neuropathy or exacerbate neurodegenerative diseases. This approach stems from understanding both how micronutrients function and how diseases develop. In inflammatory conditions, supplementation addresses increased micronutrient consumption and oxidative stress (e.g., vitamin D supplementation potentially delays dementia). Often, supplementation follows recommended daily allowances (RDAs) without individual-level verification, using higher doses initially to correct deficits. However, high vitamin C doses require caution due to potential absorption issues, oxalate kidney stones, and oxidative stress. Another perspective involves using higher doses of micronutrients to treat existing symptomatic diseases. This is well-established in some cases (e.g., thiamine for Wernicke’s encephalopathy) (Attaluri et al., 2018), but the mechanism is less clear in others (e.g., for Alzheimer’s disease, vitamin D). We either anticipate that the pathophysiology of particular micronutrients will have a beneficial influence on the development of a specific disease, or we know that the symptoms are directly caused by or associated with a fatal deficiency in micronutrient(s). One illustration of how the potential clinical effects of micronutrient delivery are typically quite challenging. It has also been demonstrated how vitamin B12 substitution affects pernicious anaemia. Administration and Vitamin B groups is more likely to have a positive effect on skin infections than on neuritis, where it is typically used as a causal therapy. In most indications, the pathophysiology of specific micronutrients justifies their prescription, which is considered an essential component of comprehensive treatment. Patients with Amyotrophic Lateral Sclerosis (ALS) experience the gradual destruction of motor neurons in the brain and spinal cord regions (Feldman et al., 2022). The disease process causes both lower motor neuron effects, which include brainstem and spinal cord muscle weakness and wasting, as well as involuntary muscle twitching. Upper motor neuron symptoms also manifest, including spasticity, exaggerated reflexes (hyperreflexia), and weakness, originating from the motor cortex. The pathological cells of ALS patients contain two significant forms of abnormal cytoplasmic structures: Bunina bodies and aggregates of TDP-43 protein (Mori et al., 2019). The complex development of ALS involves several contributing elements, including RNA processing breakdowns, protein quality control imperfections, excitotoxicity, cytoskeletal abnormalities, mitochondrial dysfunction, viral infections, and cell death through programmed pathways (apoptosis), as well as deviations in growth factor signaling and inflammatory processes.

### 13.2 Alzheimer’s disease

Alzheimer’s disease functions as the primary trigger of dementia among elderly patients. Neurotic plaques, together with amyloidβ extracellular deposits and neurofibrillary tangles, indicate the illness process. Tau is a protein that is linked to microtubules and helps them assemble, and is also related to the alterations that take place (Scheltens et al., 2021). Tau proteins are arranged into neurofibrillary tangles because of changes in their structure in AD. The development of AD has also been linked to the neuroinflammatory process, which involves elevated levels of inflammatory cytokines and amyloid-β plaques near glial cells. Patients with AD also have lower amounts of the neurotransmitter acetylcholine. Manganese disrupts the cellular balance of cholinergic neurons in certain regions, acting as a chemical stressor. Specifically, several cholinergic synaptic processes are potentially affected by manganese, including the uptake of choline before the synapse. Acetylcholine is released into the synaptic cleft in discrete packets, binds to receptors along the synapse, and is subsequently broken down by acetylcholinesterase in the synapse (Masters et al., 2015). Manganese plays a significant role in regulating choline transport systems and acetylcholine-binding proteins within astrocytes. Scientific evidence indicates that AD development and progression strongly relate to both oxidative stress and micronutrient deficiencies. Oxidative stress plays a significant role in the neurodegeneration and cognitive decline characteristics of AD. In addition to elevated homocysteine (Hcy) levels, which are linked to cognitive decline, AD patients frequently exhibit deficiencies in various vitamins (A, B1, B9, B6, B3, B12, D, C, and E) and trace elements (copper, zinc, and selenium). Vitamins B12, B9, and B6 are essential for Homocysteine metabolism, and the deficiency of specific vitamins leads to the development of hyperhomocysteinemia (Kim et al., 2018). While supplementation can decrease Hcy levels, its impact on improving cognition in mild AD is inconsistent. A deficiency in vitamin B1 is associated with cognitive decline and can be addressed through supplementation (Rajaram et al., 2019). Vitamin B12 may have the ability to inhibit tau fibrillization, while vitamin B3 potentially offers neuroprotective effects. The available evidence suggests that Vitamin E shows promising benefits, but research findings remain unclear. Some nutrients have been shown to potentially contribute to the development of Alzheimer’s Disease (AD) (Scheltens et al., 2021). Vitamin A exhibits anti-plaque formation properties, although vitamin D deficiency appears to increase AD risk due to its protective effects on brain tissue and anti-inflammatory properties. Trace elements also influence AD development. For example, elevated copper levels in the brain promote tau hyperphosphorylation and oxidative stress, contributing to the formation of neurofibrillary tangles. While zinc shortage is linked to memory and learning problems, optimum zinc levels may decrease the onset of AD. Manganese may influence glutamate modulation and astrocyte activity, potentially leading to neurotoxicity. Elderly cognitive function is positively connected with selenium.

### 13.3 Parkinson’s syndrome

Parkinson’s disease is a degenerative neurological ailment brought on by the gradual decline of dopamine-producing neurons in the basal ganglia. This neuronal loss, along with the involvement of other types of neurons, leads to a range of both movement-related (motor) and non-movement-related (non-motor) symptoms. The primary feature of Parkinson’s disease is the accumulation of alpha-synuclein (αS) protein, which forms Lewy bodies and Lewy neurites (Ye et al., 2023). PD evolution depends on both neuroinflammation and oxidative stress as additional contributors to the condition. Multiple PD pathogenesis factors result from deficits of vitamins A, D, E, B1 (thiamine), B6, C, and folate. Low vitamin C levels hinder the absorption of levodopa, while thiamine deficiency speeds up the degradation of dopaminergic neurons. By halting levodopa’s peripheral breakdown, vitamin C enhances its bioavailability and improves uptake by the central nervous system, particularly in older patients with Parkinson’s disease. Furthermore, vitamin C levels in plasma and lymphocytes are reduced in patients with PD, particularly in more advanced stages. Parkinson’s disease (PD) risk may rise if Vitamin B6 deficiency persists (Modica et al., 2023). Additionally, early olfactory problems in PD patients are linked to low consumption of thiamine and folate, vitamins vital for the proper function and control of the sense of smell. The brain benefits from vitamin D exposure because it helps minimize inflammatory responses and oxidative stress conditions. Sunlight exposure for a minimum of 15 min per week helps patients achieve optimal vitamin D blood levels. The proper levels of vitamin D led to improved motor function, along with a decreased risk of Parkinson’s disease. Regardless of age or sex, vitamin E intake has been shown to dramatically lower the incidence of Parkinson’s disease and promote dopaminergic receptor function. Compared to vitamin C and carotenoids, it provides more substantial preventive advantages. Parkinson’s disease pathogenesis is directly influenced by homocysteine levels, which contribute to the death of dopaminergic neurons (Ye et al., 2023).

### 13.4 Epilepsy syndrome

One of the potential processes of epileptogenesis is neurodegeneration induced by oxidative stress. Patients with epilepsy also showed elevated lipid peroxidation. Mitochondrial malfunction was linked to the onset of epilepsy (Moos et al., 2023). Clinical studies have demonstrated that the daily administration of 400 IU of vitamin E reduces seizure occurrence by 60% more effectively than a placebo treatment, as found through research. Research indicates that multiple vitamins, alongside related substances, result in promising results when dealing with epilepsy. Patients who take vitamin E supplements over an extended period may experience positive outcomes in reducing the frequency of recurrent seizures. Vitamin D is now being investigated in ongoing research for potential use as an additional form of therapy. High doses of vitamin B6 can successfully manage seizures that result from vitamin B6 deficiency. Studies indicate that patients experience benefits from seizure control when taking a treatment regimen that includes pyruvate and the simultaneous administration of vitamin C and vitamin E.

### 13.5 Wilson’s syndrome

WD represents an inherited disorder of metabolism that develops from defects in the ATP7B gene. Mutations within the ATP7B gene cause copper accumulation to occur in multiple body tissues, most notably the liver and brain (Telianidis et al., 2013). WD might show up as hepatic symptoms, behavioral abnormalities, intellectual disorders, and movement deficits (Parkinsonian syndromes, dyskinesias). Oral zinc administration is a well-established treatment method for WD patients with low blood copper levels. The maximum affinity exists between copper and metallothionein, a protein produced in response to hyperzincemia, as this protein helps decrease non-bound zinc levels (Cox et al., 2022). The body decreases its copper levels when zinc levels are elevated.

### 13.6 Myasthenia gravis syndrome

Myasthenia gravis (MG) is an autoimmune disease that targets the neuromuscular junctions, leading to skeletal muscle weakness (Pathak et al., 2021). The immunoglobulin G (IgG) class of antibodies is the biological basis of the illness, which targets specific postsynaptic neuromuscular junction molecules, usually nicotinic acetylcholine receptors, thereby impairing neuromuscular transmission. The presence of thyroid conditions combined with systemic lupus erythematosus, rheumatoid arthritis, and additional autoimmune disorders commonly occur among individuals with MG. Patients with MG showed noticeably reduced plasma concentrations of 25-OH vitamin D. Furthermore, it was found that patients with myasthenic crisis had lower levels than those with MG without myasthenic crisis. Scientific research suggests that vitamin D supplements hold promise in preventing myasthenic crisis attacks and highlight the role of vitamin D in the development of myasthenia gravis (MG). Vitamin B12 and folic acid levels did not significantly differ between myasthenic crisis and noncrisis periods (Yevgi and Baydar, 2022). Since managing MG necessitates long-term corticosteroid and other immunosuppressive medication, there are several side effects (infections, osteoporosis). Additionally, anemia is a common condition among female patients.

### 13.7 Huntington’s syndrome

Huntington’s disease (HD) leads to involuntary movement known as chorea, together with cognitive dementia, psychiatric symptoms, and impairments in voluntary motor control as it advances in patients (Stoker et al., 2022). HD proves its association with a CAG trinucleotide repeat expansion occurring inside the HTT genetics. This gene encodes the huntingtin protein. Through various processes, most notably oxidative stress and neuroinflammation, this mutation leads to neuronal dysfunction and cell death. In HD, oxidative stress and chronic neuroinflammation are thought to drive the development of motor problems and dementia significantly. Therefore, nutritional interventions, especially those that deliver antioxidants and neuroprotective micronutrients, are being explored as supportive therapies to slow disease progression. Specifically, Vitamin C and D, used in conjunction with conventional treatments, may help slow the progression of HD and improve balance. Furthermore, coenzyme Q10, along with vitamins B1 (thiamine), B3 (niacin), A, and E, possess significant neuroprotective properties, likely due to their support of mitochondrial function and antioxidant defenses (Rai et al., 2021). Research shows that the Mediterranean diet provides antioxidants and key micronutrients, including C, B1, B3, B6, E, selenium, and zinc, which improve quality of life and brain performance while enhancing the motor skills of HD patients. Additionally, longitudinal research has shown that zinc, selenium, and vitamin E can enhance episodic memory function over 6 years. Improving micronutrient intake, especially B vitamins, is crucial because HD patients have higher caloric needs. Additionally, following the Mediterranean diet, which is rich in antioxidants and micronutrients such as zinc, selenium, vitamins C, B1, B3, and B6, has been linked to improvements in the quality of life and cognitive and motor abilities of HD patients (Stoker et al., 2022).

### 13.8 Demyelinating diseases, including multiple sclerosis

Trimmed immune-mediated conditions, including myelin oligodendrocyte glycoprotein antibody-associated disease (MOGAD) and neuromyelitis optica spectrum disorder (NMOSDs), as well as multiple sclerosis (MS), target the brain together with the optic nerves and spinal cord (Liu et al., 2025). These disorders cause a variety of neurological symptoms and are typified by demyelination and neurodegeneration. Demyelinating lesions, also known as plaques, are associated with chronic inflammation in multiple sclerosis (MS) and are primarily caused by CD4^+^ and CD8^+^ T lymphocyte infiltrates. Current therapeutic techniques focus on immunomodulation, neuroprotection, reducing oxidative stress, and maintaining the integrity of the blood-brain barrier. Epidemiological research suggests a correlation between lower blood levels of 25-hydroxyvitamin D (25(OH)D), often associated with limited sun exposure, and an increased risk and severity of multiple sclerosis (MS). Although having enough 25(OH)D in the blood may decrease the chance of getting MS and the number of relapses, it is yet unknown if vitamin D supplementation can effectively prevent relapses. Additionally, higher homocysteine (Hcy) and decreased vitamin E levels are common in MS patients, indicating that oxidative stress may contribute to the disease’s progression (Vezzoli et al., 2023). Vitamins A and E, with their anti-inflammatory and antioxidant capabilities, may offer neuroprotective effects. Interestingly, vitamin A may also help promote myelin repair.

### 13.9 Ischemic (thrombotic and embolic) stroke

When cerebral blood vessels are blocked by thrombosis, embolism, or systemic hypoperfusion, an ischemic stroke occurs. A series of pathological events, including metabolic stress, plasma membrane disruption, intracellular content leakage, and ultimately neuronal dysfunction and cell death, is triggered by the subsequent decrease in cerebral perfusion (Anwar et al., 2024). Inflammatory reactions, acidosis, oxidative stress, cytokine-mediated cytotoxicity, complement system activation, and blood-brain barrier compromise all worsen this ischemic insult. There is growing evidence that certain micronutrients may offer protection against ischemic stroke. Belonging to a diverse group that includes vitamins B6, C, and D, beta-carotene, folic acid, and magnesium, together with potassium, may support the proper functioning of the neurovascular system. Potassium, along with magnesium and vitamins E and C, reduces the risk of ischemic stroke without increasing the likelihood of hemorrhagic stroke. Vitamin C is essential for cardiovascular health, as its anti-inflammatory, antioxidant, and endothelial-stabilizing effects can help prevent ischemic events (Xu et al., 2025). The link between elevated homocysteine levels (a stroke risk factor) and micronutrient status further emphasizes the crucial role micronutrients play in preventing cerebrovascular disease.

### 13.10 Neuropathy and myopathy

Muscle fibre dysfunction is the primary feature of myopathies, which are muscular disorders that occur without nerve innervation or defects in the neuromuscular junction. Congenital, idiopathic, metabolic, infectious, inflammatory, endocrine, toxic, and drug-induced causes are among the various etiologies of myopathies (Güler et al., 2022). Idiopathic inflammatory myopathies frequently co-occur with other autoimmune diseases, including systemic lupus erythematosus (SLE), dermatomyositis, and rheumatoid arthritis (RA) (Cutolo and Nikiphorou, 2022). Inflammatory myopathy in certain patients has also been linked to statin therapy. Furthermore, excessive alcohol use can result in acute myopathies. One of the leading causes of myopathy is nutritional deficiencies. While vitamin E deficiency is associated with neuromuscular symptoms such as ataxia, peripheral neuropathy, and proximal muscle weakness, selenium deficiency can lead to both cardiac and skeletal myopathies. Supplementing with vitamin E (usually 200 mg/day) often improves these symptoms. In situations of mitochondrial myopathy, selenium, zinc, coenzyme Q10, and vitamins B-complex, C, and E can all enhance mitochondrial activity (Rai et al., 2021).

Toxin exposure and systemic illnesses frequently result in peripheral neuropathy. Chemotherapy drugs, including taxanes, vinca alkaloids, and platinum-based agents, often cause chemotherapy-induced peripheral neuropathy (CIPN) as a side effect (Kerckhove et al., 2017). Diabetic neuropathy, especially diabetic sensory neuropathy, affects over half of individuals with long-term diabetes. Chronic alcohol use is another major contributor to neuropathy, stemming from the direct toxic effects of alcohol, nutritional deficits (especially B vitamins), and liver problems. The variable involvement of large and small nerve fibres leads to a wide range of symptoms. Vitamins B1, B6, B12, vitamin E, and copper are essential micronutrients for the health and function of the peripheral nerves. Neuropathies caused by nutritional deficiencies can appear suddenly, gradually, or over a long period, and can damage the myelin sheath or the nerve axon itself. Sometimes, both peripheral nerves and the spinal cord are impacted simultaneously (myeloneuropathy), often due to vitamin B12 or copper deficiency (Hamel and Logigian, 2023). Vitamin B1 (thiamine) deficiency has a severe impact on the nervous and cardiovascular systems. Brain effects include Wernicke encephalopathy, marked by ataxia, abnormal eye movements, and confusion. In the peripheral nervous system, damage to large fibers leads to painful sensory neuropathy and impaired mobility. This is particularly pronounced in alcohol-related neuropathy (ALN), where a lack of thiamine exacerbates alcohol’s toxicity.

While an excess of pyridoxine (vitamin B6) can paradoxically cause neuropathy, a deficiency can result in sensory neuropathy or paresthesia. Vitamin B12 (cobalamin) is crucial for the myelination of both the central and peripheral nervous systems (Mathew et al., 2024). Its deficiency leads to demyelination in the spinal cord’s dorsal and lateral columns, as well as damage to the peripheral and optic nerves. A deficiency is indicated by a serum B12 concentration of less than 150 pg/mL. A folate deficiency may exacerbate myelopathy or optic neuropathy. The primary organ affected by vitamin E deficiency is the central nervous system, but in rare instances, it can also manifest as peripheral neuropathy. Normal neurological function depends on copper’s role in cellular homeostasis, mitochondrial oxidative metabolism, and neurotransmitter biosynthesis (An et al., 2022). B vitamins are especially crucial for nerve health in diabetic polyneuropathy. Thiamine (B1) restores normal nociceptive responses by promoting nerve regeneration and guarding against oxidative stress. While cobalamin (B12) supports nerve survival, remyelination, and myelin sheath maintenance, which may result in complete nerve function recovery, pyridoxine (B6) aids in neurotransmitter synthesis and sensory nerve recovery. Furthermore, the onset and advancement of diabetic peripheral neuropathy are closely linked to vitamin D deficiency.

### 13.11 Restless legs syndrome (RLS) syndromes

Since movement produces discomfort in the legs, restless legs syndrome (RLS), a movement disorder, is characterized by an insatiable desire to move (Manconi et al., 2021). The precise etiology is unknown, but it involves hereditary factors, neurotransmitter abnormalities, and aberrant brain iron homeostasis. Restless Legs Syndrome (RLS) affects roughly 25% of pregnant women, though it typically resolves after childbirth. Research suggests a potential connection between iron deficiency and the progression or exacerbation of RLS (Manconi et al., 2021).

### 13.12 Nerve injury at the peripheral site

A peripheral nerve injury can result in loss of structural, sensory, and motor function. Because the injury causes the axon, connective tissue, and myelin sheath to rupture, the results are typically unfavorable (El Soury et al., 2021). Neurotropic B vitamins (B12, B6, B1) preserve the viability of neurons. B12 preserves myelin sheaths, B6 enhances nerve metabolism, and B1 acts as an antioxidant at the site of the injury. Peripheral neuropathy can result from a lack of these vitamins, which can cause irreversible nerve damage and pain (El Soury et al., 2021).

## 14 Conclusion

Micronutrients, particularly methyl-donor vitamins such as choline, folate, and B vitamins, have a significant impact on brain development and cognitive function through epigenetic mechanisms. It illustrates how these micronutrients affect DNA and histone methylation, thereby regulating gene expression patterns that are essential for normal neurodevelopment and mental health outcomes. Ensuring adequate intake of these nutrients during key developmental stages is crucial for mothers and young children. Deficiencies can lead to serious problems, including neural tube defects, behavioral changes, and cognitive impairments. The review also situates these findings within the broader context of gene-environment interactions, illustrating how epigenetic modifications serve as a pivotal interface through which environmental factors, such as diet, shape long-term brain health and susceptibility to neuropsychiatric and metabolic disorders. It also identifies new studies on the gut-brain-microbiota axis, suggesting that microbial metabolites influenced by diet may further contribute to epigenetic regulation of brain function, opening new avenues for therapeutic interventions. Collectively, this synthesis of current evidence advocates for targeted nutritional strategies and early interventions to optimize cognitive health across the lifespan by harnessing the epigenetic potential of micronutrient enrichment. These findings pave the way for tailored dietary approaches that prevent cognitive decline and enhance mental health through epigenetic alteration.
