# Supplementary material for: Unveiling the mechanistic nexus: how micronutrient enrichment shapes brain function, and cognitive health
Source: Front Mol Biosci. 2025 Sep 23;12:1623547. doi: 10.3389/fmolb.2025.1623547 (PMC12500463; doi:10.3389/fmolb.2025.1623547)
Supplement: Supplementary file 1 [file Table1.docx]

**Supplementary Table 1: Various crucial micronutrients with recommended doses in various lifespan, mode of action, genetic regulation and synergistic or antagonistic potential.**

| **Types of Micro Nutrients** | **Doses of Consumption** | **Role of Brain and Cognitive Development** | **Genetic regulation** | **Synergistic and Antagonastic effects** | **References** |
| --- | --- | --- | --- | --- | --- |
| Choline | Adult females 425 mg/day, Adult Males  550 mg/day | Choline availability affects brain structure development, low availability results in a Substantially reduced pool of neural progenitor cells (NPCs)  In adult higher concurrent choline intake was related to better cognitive performance, particularly in Verbal Memory (VM) and visuospatial memory (VsM), moderate choline intake may reduce the risk of low cognition (LC). | PEMT (Phosphatidylethanolamine N-Methyltransferase) is critical for endogenous choline synthesis via phosphatidylcholine production.  CHDH (Choline Dehydrogenase) variants (e.g., rs9001, rs12676) influence choline flux toward betaine synthesis versus cytidine diphosphate (CDP)-choline pathways.  BHMT (Betaine-Homocysteine Methyltransferase) converts choline-derived betaine to methionine, linking choline and folate metabolism. | Choline + Vit B : Vit B enhance cholinr availability by boostying methylation capacity.  Choline+ Zinc : Zinc supports choline kinase activity, enhancing phosphatidy;choline synthesis for cell membrane integrity.  Choline + Lutein : Enhance visual Processing  Antagonastic effects :  High dose of choline supplementation may reduce Vit K dependent Carboxylase activity.  Excessive magnesium reduces choline uptake in enterocytes by occupying cationic transporters.  In folate deficient states, choline becomes the primary methyl donor, depending hepatic reserves and increasing NAFLD risk | Poly et al., 2011  Liu et al., 2021  Zeisel, 2012  Ganz et al., 2017  Zeisel, 2017  Irvine et al., 2022  Derbyshire et al., 2020 |
| Iron | Adult Male : 8mg/ day Adult Female :18 mg/ day | Iron plays a critical role in Neuro transmitor synthesis (such as dopamine,serotonin and norepinephrin), Myelination process and cell development  Iron has been shown to improve intelligence, attention, concentration. | HFE Gene  And its mutations such as H67D lead to increase brain ion accumulation .  BTBD9 has accociation with iron concentration in specific brain regions like the ventral midbrain. | Iron shows synergistic effects with Zinc, VitC, Copper, Folate and VitB 12  And Antagonastic effects with calcium, Polyphenols. | McCann et al., 2020 Gutema et al., 2023 |
| Zinc | Adult Male: 11mg/day Adult Female: 08mg/day | Zinc is actively involved in neurogenesis ad neuronal migration, particularly in key brain regions such as the fetal cortex, hippocampus and cerebellum. Zinc contributes significantly to the structural development development and overall function of the cerebrum  Zinc is essential for learning and memory processes, promoting healthy motor development, and preserving synaptic effectiveness. Zinc is required for appropriate neurogenesis and migration, myelination, and synaptogenesis; deficiencies in zinc during infancy have been linked to delays in motor development as well as negative effects on attention span and short-term memory. | The main zinc-dependent transcription factor in charge of coordinating cellular reactions to variations in zinc availability is Metal-Responsive Transcription Factor 1 (MTF-1).  Zinc exporters (such as ZnT5/SLC30A5) that transport zinc into organelles or extracellularly and lower cytosolic zinc are encoded by SLC30A (ZnT). Importers that enhance cytosolic zinc by importing it from extracellular areas or organelles are encoded by SLC39A (ZIP). | Supplementing with zinc and vitamin A increases T-cell proliferation and cytokine production (IL-2, IFN-γ) while varying apoptosis. In hypothyroidism, zinc+selenium co-treatment raises free T3, T4, and TSH levels more effectively than individual supplementation. By improving immune cell function and cytokine modulation, zinc plus vitamin C lessens the intensity and duration of the common cold. In non-alcoholic fatty liver disease, zinc and vitamin E work together with chlorella vulgaris to reduce inflammation and improve glucose regulation. Zinc+Iron: Co-supplementing zinc and iron improves the conversion of provitamin A carotenoid to retinol.  Zinc+Copper: Zinc excess (>50 mg/day) prevents copper absorption, increasing the risk of anemia and insufficiency. Zinc+Iron: Excess zinc hinders iron uptake, while high iron dosages decrease zinc absorption. In the intestines, zinc and folic acid combine to form insoluble complexes that decrease the absorption of both minerals. Zinc+Calcium: Although the evidence is conflicting, a high calcium consumption (such as via supplements) may hinder the absorption of zinc. | Roberts et al., 2022  Georgieff et al., 2018  Choi et al., 2020  Cusick  et al., 2016  Townsend et al., 2023 |
| Vit B12 | The RDI for vitamin B12 in adults over 14 is 2.4 mcg.  Pregnant Individuals: 2.6 mcg  Breastfeeding Individuals: 2.8 mcg | DNA production, methylation processes, and the development of myelin the protective covering that surrounds nerve fibers and facilitates effective brain communication all depend on Vitamin B_12_. It is a cofactor in several catalytic processes that are necessary for the production and operation of neurotransmitters, which are the brain's chemical messengers.  Memory and attention are among the cognitive functions that Vitamin B_12_ continues to support; neurological diseases and cerebral atrophy are linked to Vitamin B_12_ deficiency. The growing brain during infancy may suffer from a Vitamin B_12_ shortage, which may result in permanent impairments in neurocognitive function. | Transcobalamin (TCN) genes: B12 transport is impacted by variations in TCN1 (transcobalamin I) and TCN2 (transcobalamin II). Fucosyltransferase 2 is encoded by the FUT2 gene, which influences B_12_ status through interactions with the gut microbiota. Different ethnic groups have different FUT2 variations. MMACHC, a gene essential for intracellular B_12_ processing, is regulated by the transcriptional regulators HCFC1 and RONIN (THAP11). A condition similar to cblC is brought on by mutations in HCFC1 or RONIN. MMACHC: The most prevalent gene mutation affecting B_12_-dependent enzyme function in cblC illness. | VitB_12_ + Follate : As a cofactor for methionine synthase, Vitamin B_12_ uses 5-methyltetrahydrofolate (5-MTHF) to convert homocysteine to methionine. This process avoids "methyl trapping" and replenishes active folate for DNA synthesis.  VitB_12_+VitB_6_: To metabolize homocysteine, B_6_, B_12_, and folate collaborate. In addition to B_12_'s function in methionine production, B_6_ helps convert homocysteine to cysteine.  VitB_12_+Iron: Megaloblastic anemia is avoided by enhancing iron's function in hemoglobin production with B_12_ and folate.  VitB_12_+VitC: There is little proof that extremely high vitamin C dosages could break down Vitamin B_12_ in food, although this depends on the situation and isn't always important.  VitB_12_+Potassium: Although there is rarely any clinical significance, high potassium levels may decrease B_12_ absorption in people with borderline B_12_ status. | Surendran et al., 2018  Padmanabhan  et al., 2017  Froese et al., 2010 |
| Vit B_9_ (Folate) | Adults: 400 mcg DFE (Dietary Folate Equivalents).  Pregnanat Woman: 600mcg -DFE  Breastfeeding Woman: 500mch DFE | Folate contributes to the maintenance of the lipids in neuronal and glial membranes and is engaged in brain methylation processes.  Early-stage maternal folate shortage has been associated with a higher incidence of congenital abnormalities, highlighting the significance of sufficient maternal consumption. Supplements of Folate used to improve memory function. Neural tube abnormalities, DNA and RNA synthesis, methylation, neuronal structure, and cognition are important words frequently linked to folate and brain development. | At an acidic pH, intestinal folate absorption is mediated by SLC46A1 (Proton-Coupled Folate Transporter). One important folate transporter is encoded by RFC1 (Reduced Folate Carrier). The efficiency of folate absorption is changed by the RFC1 80G>A polymorphism (rs1051266).  Methylenetetrahydrofolate Reductase, or  MTHFR:  In TT homozygotes, the C677T polymorphism (rs1801133) decreases enzyme activity by about 70%, raising the risk of homocysteine and NTD, particularly when folate intake is inadequate.  When paired with C677T, the A1298C polymorphism (rs1801131) worsens folate insufficiency by altering enzyme stability. | VitB9+VitB12: By giving vitamin B12-dependent methionine synthase a methyl group, folate (as 5-methyltetrahydrofolate) transforms homocysteine into methionine.  VitB9+VitB6: By using cystathionine beta-synthase to convert homocysteine to cysteine and enhancing methionine synthase activity, B6 promotes folate and B12.  VitB9+Iron: Iron promotes the formation of hemoglobin, while folate and vitamin B12 allow DNA synthesis in red blood cell precursors. Anemia due to deficiencies in any reason  VitB9+Zinc: Dietary folate is released for absorption via zinc-dependent enzymes, such as folate polyglutamate hydrolase. Proteins such as MTHFR include zinc fingers that regulate the expression of genes linked to folate.  VitB9+ B12 deficiency : Anemia is corrected by high folate (>1,000 mcg/day), however neuropathy and other neurological disorders brought on by a B12 deficiency are not prevented. | Zheng and Cantley, 2019  Coppedè, 2015  van der et al., 2006  Townsend et al., 2023  Lony et al., 2020 |
| Omega-3 Fatty acid | Adult Female : 1.32 g  Adult male1.55 g | Omega-3 fatty acids, in particular docosahexaenoic acid (DHA), are essential long-chain polyunsaturated fatty acids that are important signaling molecules and structural elements of brain cell membranes, particularly during pregnancy and infancy. The most prevalent omega-3 fatty acid in brain cell membranes is DHA. Omega-3 fatty acids continue to be crucial for learning, memory, and cognitive function throughout childhood and adolescence. A lack of DHA has been linked to mental health issues and may affect memory and learning. In the central nervous system, omega-3 fatty acids improve the way impulses are conducted at synapses. | PPARs (Peroxisome Proliferator-Activated Receptors): PPARα and PPARγ are ligands for omega-3 fatty acids, which activate genes related to lipid homeostasis and fatty acid oxidation (e.g., APOA1, LPL). Sterol Regulatory Element-Binding Proteins, or SREBPs: Omega-3 fatty acids prevent the synthesis of fatty acids by suppressing SREBP-1c, a regulator of lipogenic genes. Fatty acid desaturase gene variations, such as rs174547 in FADS1, affect how well precursor fatty acids (ALA, LA) are converted into long-chain polyunsaturated fatty acids (EPA, DHA, and AA).  PPARα L162V Polymorphism: The PPARα-V162 allele modifies reactions to omega-3 supplementation; carriers exhibit variable levels of APOA1, LPL, and PPARα expression in macrophages. | By promoting δ6-desaturase activity, which is essential for brain development, omega-3 fatty acids plus iron, zinc, B6, and E can improve the conversion of α-linolenic acid (ALA) to DHA. Erythrocyte omega-3 levels are decreased by iron shortage and restored by supplementation. In models of pregnancy-induced hypertension, omega-3 fatty acids plus vitamin B12 and folate lower homocysteine and oxidative stress, lowering the risk of preeclampsia.  Although there is little concrete data, omega-3 fatty acids and excess vitamin A may compete with omega-3s for metabolic pathways. It is possible that excessive amounts of fat-soluble vitamins could disrupt the metabolism of fats. Unbalanced Iron + Omega-3 Fatty Acid: Too much iron might worsen oxidative stress, negating the anti-inflammatory effects of omega-3. Deficiency, however, hinders the conversion of omega-3. Omega-3 Fatty Acid + High-Dose Antioxidants: Although they usually work in concert, too much antioxidant (like vitamin E) may, in some situations, reduce the anti-inflammatory benefits of omega-3-derived resolvins, but this is still poorly understood. | Roberts et al., 2022  Georgieff et al., 2018  Gómez-Pinilla, 2008  Deckelbaum , 2006  Rudkowska et al., 2009  Ahmadi et al., 2023  Kemse et al., 2014 |
| Vit-D | 400 international units (IU) for infants under 12 months, 600 IU for adults between the ages of 1 and 70, and 800 IU for those beyond 70. | Vitamin D is a fat-soluble vitamin that has significance throughout pregnancy, infancy, and childhood and may affect brain development through its function in calcium control and other mechanisms. As a neurosteroid, vitamin D interacts with the brain's vitamin D receptors to support neuronal maturation and differentiation. Because vitamin D helps brain cells produce mood-regulating neurotransmitters, it may also help sustain a pleasant mood. Cognitive decline and an elevated risk of dementia have been associated with vitamin D insufficiency in older persons. In the hippocampus, the brain's memory and learning hub, vitamin D also plays a role in the process of new neurogenesis. | In humans, vitamin D mainly controls gene expression through the nuclear transcription factor known as the vitamin D receptor (VDR) and its active metabolite 1,25-dihydroxyvitamin D3 (calcitriol). Ligand-dependent activation: VDR is bound by calcitriol, which causes conformational changes that allow VDR to attach to vitamin D response elements (VDREs) in target genes and heterodimerize with retinoid X receptor (RXR). Topologically associated domains (TADs): RNA polymerase II and chromatin-modifying enzymes are drawn to loops formed when VDR-bound enhancers interact with TSS areas within the same TAD. Calcium homeostasis: TRPV6 (calcium absorption) and CYP24A1 (encodes 24-hydroxylase, which breaks down calcitriol) are directly controlled. Chromatin accessibility: Pioneer factors (e.g., PU.1, CEBPA) that prime VDR binding cause calcitriol to increase accessibility at TSS regions within two hours and at enhancers by twenty-four hours. | Vitamin D + Vitamin K: Vitamin K activates osteocalcin and matrix Gla protein (MGP), which direct calcium to bones and prevent vascular calcification, while vitamin D improves calcium absorption. Combining supplements increases bone mineral density (BMD) and lowers the risk of fracture. Vitamin D + Calcium: Vitamin D increases the bioavailability of calcium via upregulating intestinal calcium transporters, such as TRPV6. Both neuromuscular function and bone mineralization depend on this synergy. Vitamin D + Magnesium: Hepatic 25-hydroxylase and renal 1α-hydroxylase need magnesium to transform vitamin D into its active form, calcitriol. Vitamin D metabolism is hampered by deficiency, while its effectiveness is increased by supplementation. Vitamin D + Zinc: By improving T-cell function and the synthesis of antimicrobial peptides (like cathelicidin), vitamin D and zinc work together to modify immunological responses. Inflammation and infection severity are decreased by this combination.  Vitamin D + Excess Vitamin A: Although there is currently little human data, high doses of vitamin A (retinol) may inhibit vitamin D signaling by competing with VDR for retinoid X receptor (RXR) heterodimerization. Intake of Calcium Out of Balance: Vascular Calcification: Although vitamin D aids in absorption, too much calcium without enough vitamin K can cause soft tissue calcification. Magnesium Deficiency: Low magnesium levels increase the risk of osteoporosis by preventing vitamin D activation and decreasing calcium absorption. | Gómez et al., 2008  Carlberg, 2018  Carlberg , 2022  Nurminen et al., 2019.  Townsend et al., 2023  Welty, 2023 |
| Magnesium | Adult Male: 400-420 mg  Adult Female: 310-320 | Through the activation of mechanisms (like mTOR) that promote ribosome synthesis and synaptic expansion, magnesium affects the establishment of neural networks during early brain development. H igher magnesium levels are linked to better memory, learning, and cognitive function. Magnesium has neuroprotective properties that lower oxidative stress and neuroinflammation, two factors linked to cognitive decline and neurodegenerative disorders. | TRPM6 (rs11144134): T allele linked to increased bone density but decreased serum Mg²⁺. The G allele of CASR (rs17251221) raises serum Mg²⁺ levels. Gitelman Syndrome: TRPM6 downregulation and hypomagnesemia are caused by mutations in the SLC12A3 (Na+-Cl+ cotransporter). HSH Syndrome: Serious deficit is caused by intestinal Mg2+ absorption being hindered by TRPM6 mutations. ARL15 relates fat mass and insulin sensitivity to Mg²⁺ excretion, and genetic variations alter the relationships between Mg²⁺ and metabolism. | Magnesium+Calcium : By controlling calcium transport, magnesium promotes bone mineralization and inhibits calcium accumulation in soft tissues.  Magnesium+VitD: In order to transform vitamin D into its active form (calcitriol), which improves calcium absorption, magnesium is necessary.  Magnesium+Potassium: Both control heart rhythm and nerve transmission, with magnesium helping cells retain potassium.  Magnesium+Zinc: Zinc promotes magnesium-dependent enzymatic activity, while magnesium increases zinc absorption.  Magnesium+Iron: Magnesium helps with iron use, which lowers the risk of anemia during pregnancy.  Magnesium+Excess Calcium: Vascular calcification and poor absorption of magnesium can result from a high calcium consumption without enough magnesium. Although there is no clinical evidence, magnesium+potassium imbalances in humans can interfere with electrolyte homeostasis. Magnesium+Excess Vitamin D: Hypercalcemia and soft tissue calcification can result from excessive vitamin D deficiency. | Slutsky et al., 2010  Kirkland et al., 2018  Bayle et al., 2021  Hruby et al., 2013  Sukla et al., 2024 |
| Vit E | Children aged 1-3: 6 mg daily  Kids (4–8 years old): 7 mg daily  Kids aged 9 to 13: 11 mg daily  Adults and Teens (14 years and older): 15 mg daily  Teens and women who are pregnant: 15 mg daily  Women and Teens Breastfeeding: 19 mg daily | Neural Tube Formation: In animal models, vitamin E deficiency is associated with defective blood-brain barrier formation and neural tube abnormalities, such as exencephaly, in embryos. Gene Regulation: In zebrafish and rats, vitamin E deprivation causes developmental defects by downregulating SOX10, a transcription factor necessary for sensory neuron specification and neural crest cell differentiation. Antioxidant Defense: Vitamin E protects the brain's neurons and synaptic plasticity, which are essential for memory and learning, by lowering oxidative stress. | The α-tocopherol transfer protein, which is encoded by the α-TTP (TTPA gene), preferentially binds α-tocopherol for systemic distribution. Ataxia with vitamin E deficiency (AVED) is brought on by mutations. The preponderance of α-tocopherol in plasma can be explained by the binding specificity of α-TTP, which has a 100% affinity for RRR-α-tocopherol compared to 9% for γ-tocopherol. Defense Against Degradation: Vitamin E is protected from CYP450-mediated ω-hydroxylation by α-TTP binding, which lowers catabolism. CYP4F2/CYP3A4: CYP4F2 catalyzes the side-chain ω-hydroxylation of vitamin E, which starts the β-oxidation process. Tocopherols are converted to carboxyethyl-hydroxychroman (CEHC) metabolites via CYP3A4. Hepatocytes produce 90% less CEHC when ketoconazole is used as an inhibitor. Peroxisomal 3-ketoacyl-CoA thiolase, or SCP-x (Sterol Carrier Protein-x), is essential for the β-oxidation of vitamin E metabolites. | Vitamin E+VitC: Vitamin C restores the antioxidant capacity of damaged vitamin E. Vitamin E+VitA: Vitamin A: Together, vitamins E and A boost overall antioxidant capacity, lower inflammation, and promote brain and immune function. Vitamin E and selenium work together to strengthen antioxidant enzyme systems, such as glutathione peroxidase, which improves cells' and tissues' resistance to oxidative damage. Vitamin E and zinc: Together with vitamin C, vitamin A, and selenium, vitamin E helps to keep antioxidant enzyme activity high, which reduces oxidative stress and boosts immunity.  Vitamin E+Iron: Consuming too much iron might hinder the absorption of vitamin E, and vice versa. To prevent either micronutrient's effectiveness from being diminished, it is advised to take iron and vitamin E pills at distinct times. Vitamin E+VitK: Vitamin E may counteract vitamin K activity, which could have an impact on blood coagulation, particularly when taken in large dosages. Those who are on anticoagulant medication or who are vitamin K deficient should be especially aware of this interaction. VitD+VitE: Although the clinical importance is yet unknown, there is some evidence that vitamin E may have antagonistic effects with vitamin D at high doses. Both are fat-soluble, and when eaten in excess, they may compete for absorption. | Lakhan et al., 2021  Fata et al., 2014  Rutjes et al., 2018  Galmés et al., 2018  Traber et al., 2021  Lakhan et al., 2021 |
| Iodine | Every day, dults require 140 micrograms (μg) of iodine. | Synthesis of Thyroid Hormones  The production of thyroid hormones (T3 and T4), which control brain development in fetuses and the first few months of life, depends on iodine. Important processes for brain connection and cognitive function, neurogenesis, myelination, and synaptogenesis, are influenced by these hormones. Cognitive development in childhood: At the population level, a little iodine shortage lowers IQ by 10–15 points. When children with deficiencies receive supplements, their cognitive scores increase, especially in thinking and memory. Effects of structure: In older adults, low iodine intake is associated with greater brain volume shrinkage, which may have long-term neurological effects. During pregnancy and the early years of life, iodine is essential. Early pregnancy-related maternal hypothyroxinemia (low thyroid hormone levels) impairs fetal brain development and results in irreversible cognitive impairments. | Thyroid hormone synthesis depends on NKX2-1/TTF1, which directly controls the expression of thyroglobulin (Tg) and genes related to iodide uptake (such as sodium-iodide symporter, NIS). FOXE1: A forkhead transcription factor that suppresses thyroid-specific genes in non-thyroid tissues and is essential for the development of the thyroid gland. The sodium-iodide symporter, which is encoded by SLC5A5 (NIS), is in charge of iodine uptake into thyroid cells. TSH and transcription factors like NKX2-1 strictly control its expression. The hydrogen peroxide needed for iodide oxidation is produced by DUOX2. These mutations affect the production of hormones and are associated with congenital hypothyroidism. Genes linked to AITD: Genetic predisposition and environmental iodine levels are linked by polymorphisms in HLA-DR, CTLA-4, and PTPN22, which increase vulnerability to AITD under high iodine intake. Mutations in BRAF: Although BRAFV600E is mainly carcinogenic, it may also have an indirect impact on iodide metabolism by changing the differentiation of thyroid cells. | Iodine+Iron: Thyroid peroxidase (TPO) activity is hampered by iron insufficiency, which lowers iodine usage. Co-supplementation lowers the frequency of goiter and enhances thyroid hormone synthesis. Iodine+Selenium: During iodine metabolism, thyroid cells are shielded from oxidative damage by selenium-dependent enzymes, such as glutathione peroxidase. Iodine+VitA: By improving iodine absorption and thyroglobulin production, vitamin A lowers the incidence of goiter. Iodine+Zinc: Although there is little evidence of a direct link, zinc insufficiency may make iodine deficit worse by affecting the production of thyroid hormones.  Iodine+Excess Iron: In genetically susceptible people, high iron levels may exacerbate thyroid autoimmunity by amplifying reactive oxygen species (ROS) during iodine metabolism. Iodine+Excess Selenium: By changing deiodinase activity, excessive selenium consumption in iodine-deficient populations may inhibit thyroid function. | Bailote et al., 2022  Espadinha et al., 2009  Waśniowska et al., 2025  Hess, 2010  Rigutto, 2023 |
| Selenium | 55 mcg per day for adults (19 years and older).  Children: The recommended dosages rise with age, from 20 mcg for kids aged 1-3 to 40 mcg for kids aged 9-13.  Pregnant Women: 60 mcg each day.  Women: 70 mcg each day. | Selenoproteins are essential for healthy brain growth. According to research on animals, serious neurodevelopmental abnormalities, such as reduced neuronal survival, hypoplasia, and even early mortality, result from the lack or genetic disruption of important selenoproteins (such as SELENOP, GPX4, and SELENOT). Rare mutations that impact the production of selenoprotein in humans cause neurological symptoms such as hearing loss, seizures, and cognitive and motor impairments. According to epidemiological research, selenium levels and cognitive function are positively correlated, and a shortage in selenium is associated with cognitive decline and a higher risk of neurodegenerative illnesses including Parkinson's and Alzheimer's. Cognitive impairment is linked to the malfunctioning of selenium proteins, including GPX4, SELENOP, SELENOK, and SELENOT, which are strongly expressed in brain regions related to cognition and memory. | Selenoproteins Are Regulated Hierarchically A tiered expression pattern is determined by Selenium status:  Stress-related selenoproteins, such as GPX1, SELENOW, and SELENOH, exhibit marked downregulation and are extremely vulnerable to selenium deprivation. Under low selenium environments, housekeeping selenium proteins (such as TXNRD1 and DIO2) stay comparatively steady.  SECIS Components and Trans-Acting Elements  For selenocysteine insertion, UGA codon recoding is made possible by the SECIS region in the 3'-UTR of selenoprotein mRNA.  SBP2 and eEFSec facilitate selenocysteine incorporation by binding SECIS and recruiting tRNA[Ser]Sec. NMD, or nonsensical-mediated decay: A lack of selenium causes NMD to break down selenoprotein mRNAs with ineffective Sec incorporation. Selenoproteins linked to stress are specifically targeted by this. Effectiveness of Translation: Low selenium causes ribosome stalling at UGA codons, which slows down Sec insertion, decreasing protein synthesis and raising mRNA turnover. Selenium affects non-selenoprotein genes related to oxidative stress (DDIT3, for example) and extracellular matrix architecture through epigenetic and transcriptional modulation. | Iodine is a structural element of thyroid hormones, and selenium-dependent enzymes (such as iodothyronine deiodinases) transform them into active forms. Thyroid equilibrium is disturbed by deficiencies in either. Selenium+VitE: Selenium and vitamin E work together to prevent oxidative damage to cell membranes through the action of glutathione peroxidase. When taken together, vitamin E improves organ protection and lessens the symptoms of selenium shortage. Zinc and Selenium: Although they are frequently hostile (see below), zinc and selenium work together to support DNA repair processes. Both lessen the risk of cancer by mitigating oxidative DNA damage. Cobalt increases the metabolic activity of selenium, and vitamin B6 facilitates the conversion of selenomethionine into glutathione peroxidase. | Solovyev, 2015  Pitts et al., 2022  Batyrova et al., 2025  Cardoso et al., 2018  Hoyos et al., 2023  Lammi and Qu, 2018  Zhang et al., 2022  Yildiz et al., 2019  Gui et al., 2022  Schrauzer, 2009  Saremi et al., 2024  Sajedi et al., 2011 |
| Copper | Adults aged 19 and up: 900 mcg daily.  Children aged 4–8: 440 mcg daily.  Children aged 9–13: 700 mcg daily.  Teens aged 14–18: 890 mcg daily.  Women who are pregnant: 1,000 mcg daily.  1,300 mcg each day for women who are nursing. | As a cofactor for enzymes involved in myelination, redox equilibrium, and neurotransmitter synthesis, copper is essential for healthy central nervous system development. By modifying AMPA and GABA receptors, increasing synaptic plasticity, and encouraging the anchoring of AMPA receptors to postsynaptic membranes via PSD-95 protein accumulation, it directly affects neurotransmission in synapses and synaptic vesicles. The locus coeruleus, a part of the brain that controls arousal and alertness, needs adequate copper levels to produce norepinephrine. In older populations, slower cognitive decline is correlated with higher brain copper levels. Research indicates that higher levels of copper in the mid-frontal and inferior temporal regions are linked to better working memory, episodic memory, and global cognition. Learning and memory formation are supported by copper's function in preserving synaptic efficiency and long-term potentiation (LTP) mechanisms. | Recoding of UGA stop codons: A selenocysteine insertion sequence (SECIS) in the 3′-UTR of selenoprotein mRNAs and a specific tRNA ([Ser]Sec) are necessary for selenocysteine (Sec) incorporation.  Transcriptional prioritization: Essential selenoproteins (such as glutathione peroxidase 1 and thioredoxin reductase 1) are prioritized during selenium deprivation, whilst other selenoproteins (such as selenoproteins H, I, and W) are downregulated.  NMD, or nonsense-mediated decay: Selenoprotein mRNAs with misread Sec codons experience NMD due to low selenium, which lowers their levels.  Regulation of microRNA (miRNA): miR-185 targets selenophosphate synthetase 2 and glutathione peroxidase 2.  Selenoprotein K is suppressed by miR-544a, whereas selenium inhibits apoptosis by modulating miR-125a.   Selenium-sensitive long non-coding RNAs (lncRNAs) affect p53 pathways, Wnt/β-catenin signaling, and stress responses. | Iron and Copper: Due to its function in ferroxidase activity, which promotes iron oxidation and hemoglobin inclusion, copper is necessary for iron consumption. On the other hand, consuming too much copper might cause a dual relationship by competing with iron for absorption. Copper and Zinc: Although antagonistic at high concentrations (see below), copper and zinc can work in concert in certain metabolic situations, such as enzyme activity and immunological function. Calcium and Vitamin D: Copper and calcium and vitamin D work together to support bone health because deficiencies in copper make bone problems related to calcium metabolism worse. Sodium and Selenium: Copper works in concert with sodium and selenium to support metabolic functions such as electrolyte homeostasis and antioxidant defense.  Copper and Zinc: While too much copper prevents zinc uptake, a high zinc diet causes mucosal proteins to bind copper, decreasing its absorption. Iron and Copper: Consuming too much iron inhibits the intestinal absorption of copper. Vitamins C and B12: Excessive amounts of these vitamins may affect the body's ability to retain or use copper. | Agarwal et al., 2022  Opazo et al., 2014.  Lammi and Qu, 2018  Cui et al. 2022  Gombart et al., 2020  Liu et al., 2024 |

**References (Supplementary Table 1)**

Agarwal, P., Ayton, S., Agrawal, S., Dhana, K., Bennett, D. A., Barnes, L. L., Leurgans, S. E., Bush, A. L. & Schneider, J. A. (2022). Brain copper may protect from cognitive decline and Alzheimer’s disease pathology: a community-based study. *Molecular psychiatry*, *27*(10), 4307-4313.

Ahmadi, A.R., Shirani, F., Abiri, B., Siavash, M., Haghighi, S., Akbari, M. (2023). Impact of omega-3 fatty acids supplementation on the gene expression of peroxisome proliferator-activated receptors-γ, α and fibroblast growth factor-21 serum levels in patients with various presentations of metabolic conditions: A GRADE assessed systematic review and dose–response meta-analysis of clinical trials. Front in Nut, 10:1202688. doi: 10.3389/fnut.2023.1202688.

Bailote, H.B., Linhares, D., Carvalho, C., Prazeres, S., Rodrigues, A.S., Garcia, P. (2022). Iodine intake and related cognitive function impairments in elementary schoolchildren. Biology, 11(10):1507. doi: 10.3390/biology11101507.

Batyrova, G., Taskozhina, G., Umarova, G., Umarov, Y., Morenko, M., Iriskulov, B., Kudabayeva, K., Bazargaliyev, Y. (2025). Unveiling the role of selenium in child development: Impacts on growth, neurodevelopment and immunity. J. Cli. Med. 14(4):1274. doi: 10.3390/jcm14041274.

Bayle, D., Coudy-Gandilhon, C., Gueugneau, M., Castiglioni, S., Zocchi, M., Maj-Zurawska, M., Palinska-Saadi, A., Mazur, A., Béchet, D., Maier, J.A. (2021). Magnesium deficiency alters expression of genes critical for muscle magnesium homeostasis and physiology in mice. Nutrients, 13(7):2169. doi: 10.3390/nu13072169.

Cardoso, B.R., Szymlek-Gay, E.A., Roberts, B.R., Formica, M., Gianoudis, J., O’Connell, S., Nowson, C.A., Daly, R.M. (2018). Selenium status is not associated with cognitive performance: A cross-sectional study in 154 older Australian adults. Nutrients, 10(12):1847. doi: 10.3390/nu10121847.

Carlberg, C. (2018). Vitamin D genomics: From in vitro to in vivo. Frontiers in Endocrinology, 9:250. doi: 10.3389/fendo.2018.00250.

Carlberg, C. (2022). Vitamin D and its target genes. Nutrients, 14(7):1354. doi: 10.3390/nu14071354.

Choi, S., Hong, D.K., Choi, B.Y., Suh, S.W. (2020). Zinc in the brain: Friend or foe? Int J Mol Sci. 21(23):8941. doi: 10.3390/ijms21238941.

Coppedè, F. (2015). The genetics of folate metabolism and maternal risk of birth of a child with Down syndrome and associated congenital heart defects. Front. in Gent. 6:223. doi: 10.3389/fgene.2015.00223.

Cui, X., He, H., Hu, S., Zhang, B., Cai, H. (2022). Synergistic interaction between copper and nitrogen-uptake, translocation, and distribution in rice plant. Plants, 11(19):2612. doi: 10.3390/plants11192612.

Cusick, S.E. and Georgieff, M.K. (2016). The role of nutrition in brain development: the Golden opportunity of the first 1000 days brain development in late fetal and early postnatal life. J Pediatr. 175:16–21. https://doi.org/10.1016/j.jpeds.2016.05.013

Deckelbaum, R.J., Worgall, T.S., Seo, T. (2006). n-3 fatty acids and gene expression. American J. Cli Nut. 83(6 Suppl):1520S-1525S. doi: 10.1093/ajcn/83.6.1520S.

Derbyshire, E., Obeid, R. (2020). Choline, neurological development and brain function: A systematic review focusing on the first 1000 days. Nutrients 12(6):1731. doi: 10.3390/nu12061731.

Espadinha, C., Santos, J.R., Sobrinho, L.G., Bugalho, M.J. (2009). Expression of iodine metabolism genes in human thyroid tissues: Evidence for age and BRAFV600E mutation dependency. Clinical Endocrinology (Oxford), 70(4):629-635. doi: 10.1111/j.1365-2265.2008.03376.x.

Fata, G.L., Weber, P., Mohajeri, M.H. (2014). Effects of vitamin E on cognitive performance during ageing and in Alzheimer’s disease. Nutrients, 6(12):5453-5472. doi: 10.3390/nu6125453.

Froese, D.S., Gravel, R.A. (2010). Genetic disorders of vitamin B₁₂ metabolism: Eight complementation groups--eight genes. Expert Review of Molecular Medicine, 12:e37. doi: 10.1017/S1462399410001651.

Galmés, S., Serra, F., Palou, A. (2018). Vitamin E metabolic effects and genetic variants: A challenge for precision nutrition in obesity and associated disturbances. Nutrients, 10(12):1919. doi: 10.3390/nu10121919.

Ganz, A.B., Cohen, V.V., Swersky, C.C., Stover, J., Vitiello, G.A., Lovesky, J., Chuang, J.C., Shields, K., Fomin, V.G., Lopez, Y.S., Mohan, S., Ganti, A., Carrier, B., Malysheva, O.V., Caudill, M.A. (2017). Genetic variation in choline-metabolizing enzymes alters choline metabolism in young women consuming choline intakes meeting current recommendations. Int J Mol Sci. 18(2):252. doi: 10.3390/ijms18020252.

Georgieff, M.K., Ramel, S.E., Cusick, S.E. (2018). Nutritional influences on brain development. Acta Paediatrica, 107(8):1310-1321. doi: 10.1111/apa.14287.

Gombart, A.F., Pierre, A., Maggini, S. (2020). A review of micronutrients and the immune system: Working in harmony to reduce the risk of infection. Nutrients, 12(1):236. doi: 10.3390/nu12010236.

Gómez-Pinilla, F. (2008). Brain foods: The effects of nutrients on brain function. Nature Reviews Neuroscience, 9(7):568-578. doi: 10.1038/nrn2421.

Gui, J.Y., Rao, S., Huang, X., Liu, X., Cheng, S., Xu, F. (2022). Interaction between selenium and essential micronutrient elements in plants: A systematic review. Science of the Total Environment, 853:158673. doi: 10.1016/j.scitotenv.2022.158673.

Gutema, B.T., Sorrie, M.B., Megersa, N.D., Yesera, G.E., Yeshitila, Y.G., Pauwels, N.S., De Henauw, S., Abbeddou, S. (2023). Effects of iron supplementation on cognitive development in school-age children: Systematic review and meta-analysis. PLoS One. 18(6):e0287703. doi: 10.1371/journal.pone.0287703.

Hess, S.Y. (2010). The impact of common micronutrient deficiencies on iodine and thyroid metabolism: The evidence from human studies. Best Practice & Research Clinical Endocrinology & Metabolism, 24(1):117-132. doi: 10.1016/j.beem.2009.08.012.

Hoyos, B.S., Hernandez-Tenorio, F., Miranda, A.M., Villanueva-Mejía, D.F., Sáez, A.A. (2023). Systematic analysis of genes related to selenium bioaccumulation in microalgae: A review. Biology (Basel), 12(5):703. doi: 10.3390/biology12050703.

Hruby, A., McKeown, N.M., Song, Y., Djoussé, L. (2013). Dietary magnesium and genetic interactions in diabetes and related risk factors: A brief overview of current knowledge. Nutrients, 5(12):4990-5011. doi: 10.3390/nu5124990.

Irvine, N., England-Mason, G., Field, C.J., Dewey, D., Aghajafari, F. (2022). Prenatal folate and choline levels and brain and cognitive development in children: A critical narrative review. Nutrients 14(2):364. doi: 10.3390/nu14020364.

Kemse, N.G., Kale, A.A., Joshi, S.R. (2014). A combined supplementation of omega-3 fatty acids and micronutrients (folic acid, vitamin B12) reduces oxidative stress markers in a rat model of pregnancy-induced hypertension. PLoS ONE, 9(11):e111902. doi: 10.1371/journal.pone.0111902.

Kirkland, A.E., Sarlo, G.L., Holton, K.F. (2018). The role of magnesium in neurological disorders. Nutrients, 10(6):730. doi: 10.3390/nu10060730.

Lakhan, R., Sharma, M., Batra, K., Beatty, F.B. (2021). The role of vitamin E in slowing down mild cognitive impairment: A narrative review. Healthcare (Basel), 9(11):1573. doi: 10.3390/healthcare9111573.

Lammi, M.J., Qu, C. (2018). Selenium-related transcriptional regulation of gene expression. Int. J. Mol Sci. 19(9):2665. doi: 10.3390/ijms19092665.

Liu, L., Qiao, S., Zhuang, L., Xu, S., Chen, L., Lai, Q., Wang, W. (2021a). Choline intake correlates with cognitive performance among elder adults in the United States. Behavl. Neuro. 2021:2962245. doi: 10.1155/2021/2962245.

Liu, Y., Qian, K., Shi, X., Jing, Y., He, H., Li, Y., Li, D., Wang, S. (2024). Synergistic effects of nutrients on musculoskeletal health in gerontology: Understanding the combined impact of macronutrients and micronutrients. Nutrients, 16(11):1640. doi: 10.3390/nu16111640.

Lyon, P., Strippoli, V., Fang, B., Cimmino, L. (2020). B vitamins and one-carbon metabolism: Implications in human health and disease. Nutrients, 12(9):2867. doi: 10.3390/nu12092867.

McCann, S., Perapoch Amadó, M., Moore, S.E. (2020). The role of iron in brain development: A systematic review. Nutrients 12(7):2001. doi: 10.3390/nu12072001.

Nurminen, V., Seuter, S., Carlberg, C. (2019). Primary vitamin D target genes of human monocytes. Frontiers in Physiology, 10:194. doi: 10.3389/fphys.2019.00194.

Opazo, C.M., Greenough, M.A., Bush, A.I. (2014). Copper: From neurotransmission to neuroproteostasis. Front in Age Neurosci 6:143. doi: 10.3389/fnagi.2014.00143.

Padmanabhan, S., Jost, M., Drennan, C.L., Elías-Arnanz, M. (2017). A new facet of vitamin B12: Gene regulation by cobalamin-based photoreceptors. Annual Review of Biochemistry, 86:485-514. doi: 10.1146/annurev-biochem-061516-044500.

Pitts, M.W., Hoffmann, P.R., Schomburg, L. (2022). Editorial: Selenium and selenoproteins in brain development, function, and disease. Frontiers in Neuroscience, 15:821140. doi: 10.3389/fnins.2021.821140.

Poly, C., Massaro, J.M., Seshadri, S., Wolf, P.A., Cho, E., Krall, E., Jacques, P.F., Au, R. (2011). The relation of dietary choline to cognitive performance and white-matter hyperintensity in the Framingham Offspring Cohort. Ame. J. Cli. Nutri. 94(6):1584-1591. doi: 10.3945/ajcn.110.008938.

Rigutto-Farebrother, J. (2023). Optimizing growth: The case for iodine. Nutrients, 15(4):814. doi: 10.3390/nu15040814.

Roberts, M., Tolar-Peterson, T., Reynolds, A., Wall, C., Reeder, N., Rico Mendez, G. (2022). The effects of nutritional interventions on the cognitive development of preschool-age children: A systematic review. Nutrients 14(3):532. doi: 10.3390/nu14030532.

Rudkowska, I., Garenc, C., Couture, P., Vohl, M.C. (2009). Omega-3 fatty acids regulate gene expression levels differently in subjects carrying the PPARalpha L162V polymorphism. Genes & Nutrition, 4(3):199-205. doi: 10.1007/s12263-009-0129-2.

Rutjes, A.W., Denton, D.A., Di Nisio, M., Chong, L.Y., Abraham, R.P., Al-Assaf, A.S., Anderson, J.L., Malik, M.A., Vernooij, R.W., Martínez, G., Tabet, N., McCleery, J. (2018). Vitamin and mineral supplementation for maintaining cognitive function in cognitively healthy people in mid and late life. Cochrane Database of Systematic Reviews, 12(12):CD011906. doi: 10.1002/14651858.CD011906.pub2.

Sajedi, N.A., Ardakani, M.R., Madani, H., Naderi, A., Miransari, M. (2011). The effects of selenium and other micronutrients on the antioxidant activities and yield of corn (Zea mays L.) under drought stress. Physiol. and Mol Biol of Pln. 17(3):215-222. doi: 10.1007/s12298-011-0067-5.

Saremi, N., Keyvanshokooh, S., Mousavi, S.M., Mohammadiazarm, H. (2024). Synergistic effects of dietary selenium nanoparticles and vitamin C improve growth performance, immune response, and antioxidant status of juvenile common carp (Cyprinus carpio). J. Tra. Ele. in Med. and Biol. 86:127530. doi: 10.1016/j.jtemb.2024.127530.

Schrauzer, G.N. (2009). Selenium and selenium-antagonistic elements in nutritional cancer prevention. Critical Reviews in Biotechnology, 29(1):10-17. doi: 10.1080/07388550802658048.

Slutsky, I., Abumaria, N., Wu, L.J., Huang, C., Zhang, L., Li, B., Zhao, X., Govindarajan, A., Zhao, M.G., Zhuo, M., Tonegawa, S., Liu, G. (2010). Enhancement of learning and memory by elevating brain magnesium. Neuron, 65(2):165-177. doi: 10.1016/j.neuron.2009.12.026.

Solovyev, N.D. (2015). Importance of selenium and selenoprotein for brain function: From antioxidant protection to neuronal signalling. J. Inorg. Biochem. 153:1-12. doi: 10.1016/j.jinorgbio.2015.09.003.

Shukla, V., Parvez, S., Fatima, G., Singh, S., Magomedova, A., Batiha, G. E. S., Alexiou, A, Papadakis, M. & Hadi, N. (2024). Micronutrient interactions: Magnesium and its synergies in maternal–fetal health. *Food Science & Nutrition*, *12*(10), 6913-6928.

Surendran, S., Adaikalakoteswari, A., Saravanan, P., Shatwaan, I.A., Lovegrove, J.A., Vimaleswaran, K.S. (2018). An update on vitamin B12-related gene polymorphisms and B12 status. Genes & Nutrition, 13:2. doi: 10.1186/s12263-018-0591-9.

Townsend, J.R., Kirby, T.O., Sapp, P.A., Gonzalez, A.M., Marshall, T.M., Esposito, R. (2023). Nutrient synergy: Definition, evidence, and future directions. Frontiers in Nutrition 10:1279925. doi: 10.3389/fnut.2023.1279925.

Traber, M.G. (2021). Vitamin E: Necessary nutrient for neural development and cognitive function. Proceedings of the Nutrition Society, 80(3):319-326. doi: 10.1017/S0029665121000914.

Van der Linden, I.J., Afman, L.A., Heil, S.G., Blom, H.J. (2006). Genetic variation in genes of folate metabolism and neural-tube defect risk. Proceedings of the Nutrition Society, 65(2):204-215. doi: 10.1079/PNS2006493.

Waśniowska, J., Piątkowska, E., Pawlicki, P., Smoleń, S., Kopeć, A., Dyląg, A., Krzemińska, J., Koronowicz, A. (2025). Comparative analysis of iodine levels, biochemical responses, and thyroid gene expression in rats fed diets with kale biofortified with 5,7-diiodo-8-quinolinol. International Journal of Molecular Sciences, 26(2):822. doi: 10.3390/ijms26020822.

Welty, F.K. (2023). Omega-3 fatty acids and cognitive function. Current Opinion in Lipidology, 34(1):12-21. doi: 10.1097/MOL.0000000000000862.

Yildiz, A., Kaya, Y., Tanriverdi, O. (2019). Effect of the interaction between selenium and zinc on DNA repair in association with cancer prevention. Journal of Cancer Prevention, 24(3):146-154. doi: 10.15430/JCP.2019.24.3.146.

Zeisel, S.H. (2012). Diet-gene interactions underlie metabolic individuality and influence brain development: Implications for clinical practice derived from studies on choline metabolism. Anl of Nutri and Metabol. 60(Suppl. 3):19-25. doi: 10.1159/000337869.

Zeisel, S.H. (2017). Choline, other methyl-donors and epigenetics. Nutrients 9(5):445. doi: 10.3390/nu9050445.

Zhang, Y., Jin, J., Huang, B., Ying, H., He, J., Jiang, L. (2022). Selenium metabolism and selenoproteins in prokaryotes: A bioinformatics perspective. Biomolecules, 12(7):917. doi: 10.3390/biom12070917.

Zheng, Y., Cantley, L.C. (2019). Toward a better understanding of folate metabolism in health and disease. J Exp Med, 216(2):253-266. doi: 10.1084/jem.20181413.
